# Cardiorenal effects of angiotensin-converting enzyme inhibitors and angiotensin receptor blockers among people underrepresented in trials: analysis of routinely collected data with emulation of a reference trial (ONTARGET)

**DOI:** 10.1093/aje/kwae137

**Published:** 2024-06-18

**Authors:** Paris J Baptiste, Angel Y S Wong, Anna Schultze, Catherine M Clase, Clémence Leyrat, Elizabeth Williamson, Emma Powell, Johannes F E Mann, Marianne Cunnington, Koon Teo, Shrikant I Bangdiwala, Peggy Gao, Laurie Tomlinson, Kevin Wing

**Affiliations:** Department of Non-communicable Disease Epidemiology, London School of Hygiene & Tropical Medicine, London WC1E 7HT, United Kingdom; Centre for Primary Care, Wolfson Institute of Population Health, Queen Mary University of London, London E1 4NS, United Kingdom; Department of Non-communicable Disease Epidemiology, London School of Hygiene & Tropical Medicine, London WC1E 7HT, United Kingdom; Department of Non-communicable Disease Epidemiology, London School of Hygiene & Tropical Medicine, London WC1E 7HT, United Kingdom; Department of Medicine, Faculty of Health Sciences, McMaster University, Hamilton, ON L8N 3Z5, Canada; Department of Health Research Methods, Evidence, and Impact, Faculty of Health Sciences, McMaster University, Hamilton, ON L8S 4L8, Canada; Department of Medical Statistics, London School of Hygiene & Tropical Medicine, London WC1E 7HT, United Kingdom; Department of Medical Statistics, London School of Hygiene & Tropical Medicine, London WC1E 7HT, United Kingdom; Department of Non-communicable Disease Epidemiology, London School of Hygiene & Tropical Medicine, London WC1E 7HT, United Kingdom; Department of Medicine 4, Faculty of Medicine, Friedrich Alexander University, 91054 Erlangen, Germany; KfH Kidney Centre, 80687 München-Schwabing, Germany; Population Health Research Institute, McMaster University, Hamilton, ON L8L 2X2, Canada; Analysis Group Inc., London EC2R 7HJ, United Kingdom; Department of Health Research Methods, Evidence, and Impact, Faculty of Health Sciences, McMaster University, Hamilton, ON L8S 4L8, Canada; Population Health Research Institute, McMaster University, Hamilton, ON L8L 2X2, Canada; Department of Health Research Methods, Evidence, and Impact, Faculty of Health Sciences, McMaster University, Hamilton, ON L8S 4L8, Canada; Population Health Research Institute, McMaster University, Hamilton, ON L8L 2X2, Canada; Population Health Research Institute, McMaster University, Hamilton, ON L8L 2X2, Canada; Department of Non-communicable Disease Epidemiology, London School of Hygiene & Tropical Medicine, London WC1E 7HT, United Kingdom; Department of Non-communicable Disease Epidemiology, London School of Hygiene & Tropical Medicine, London WC1E 7HT, United Kingdom

**Keywords:** trial emulation, trial replication, cardiovascular disease, trial generalizability, benchmarking, electronic health records, pharmacoepidemiology, real-world evidence

## Abstract

Cardiovascular disease is a leading cause of death globally. Angiotensin-converting enzyme inhibitors (ACEi) and angiotensin receptor blockers (ARB), compared in the ONTARGET trial (Ongoing Telmisartan Alone and in Combination with Ramipril Global Endpoint Trial), each prevent cardiovascular disease. However, trial results may not be generalizable, and their effectiveness in underrepresented groups is unclear. Using trial emulation methods within routine-care data to validate findings, we explored the generalizability of ONTARGET results. For people prescribed an ACEi/ARB in the UK Clinical Practice Research Datalink GOLD dataset from January 1, 2001, to July 31, 2019, we applied trial criteria and propensity-score methods to create an ONTARGET trial-eligible cohort. Comparing ARB with ACEi, we estimated hazard ratios for the primary composite trial outcome (cardiovascular death, myocardial infarction, stroke, or hospitalization for heart failure) and secondary outcomes. Because the prespecified criteria were met, confirming trial emulation, we then explored treatment heterogeneity among 3 trial-underrepresented subgroups: females, persons aged ≥75 years, and those with chronic kidney disease. In the trial-eligible population (*n* = 137 155), results for the primary outcome demonstrated similar effects of ARB and ACEi (hazard ratio = 0.97; 95% CI, 0.93-1.01), meeting the prespecified validation criteria. When extending this outcome to trial-underrepresented groups, similar treatment effects were observed by sex, age, and chronic kidney disease. This suggests that ONTARGET trial findings are generalizable to trial-underrepresented subgroups.

This article is part of a Special Collection on Pharmacoepidemiology.

## Introduction

Cardiovascular disease is a leading cause of death globally, with older people and those with chronic kidney disease (CKD) at particularly high risk.^1^ Medications used to prevent cardiovascular events are prescribed based on evidence from randomized controlled trials. However, there is uncertainty about whether trial evidence is generalizable to all patient groups, because trials often restrict inclusion to younger patients with fewer comorbid conditions^2^^,^^3^ and because trial patients are likely to have better adherence and monitoring. Observational studies using routinely collected health-care data can use trial-emulation methods to benchmark findings against those from existing randomized trials, sometimes referred to as reference trials or index trials.^4^^-^^10^ When similar findings are observed, we have more confidence that sources of bias and confounding are minimized, and aided by a large sample size and more diverse population, we can then examine treatment effectiveness in trial-underrepresented or excluded groups.^11^

ONTARGET (Ongoing Telmisartan Alone and in Combination with Ramipril Global Endpoint Trial) was a large global trial with 25 620 patients and a follow-up period of 3.5-5.5 years that compared the cardiovascular effects of an angiotensin receptor II blocker (ARB) (telmisartan) with those of an angiotensin-converting enzyme inhibitor (ACEi) (ramipril) among patients who had vascular disease or high-risk diabetes.^12^^,^^13^ Ramipril had previously been shown, in comparison with placebo, to reduce the composite outcome of myocardial infarction (MI), stroke, or cardiovascular death by 22% (95% CI, 14-30).^14^ The findings of the ONTARGET trial of noninferiority for telmisartan versus ramipril led to telmisartan’s licensing for cardiovascular event reduction in 2009^15^ and were a major contribution to the perception of equivalent treatment effectiveness for ARB and ACEi. However, the relative effectiveness of ARB and ACEi for patients not included or underrepresented in ONTARGET remains uncertain.

Our aims in this study were to demonstrate whether the primary and secondary outcome results of the ONTARGET trial could be replicated in routinely collected UK data and, if so, to examine treatment effects in females, persons aged ≥75 years, and those with CKD—all groups that were underrepresented in ONTARGET.

## Methods

### The reference trial (ONTARGET)

The primary objective of the ONTARGET trial was to demonstrate the noninferiority of 80 mg of telmisartan daily (ARB) versus 10 mg of ramipril daily (ACEi) in reduction of cardiovascular events among patients who had vascular disease or high-risk diabetes but did not have heart failure. The secondary objective was to determine whether a combination of the two medications was superior to ramipril alone.^12^ Patients were eligible if they were aged ≥55 years and had a history of either coronary artery, peripheral artery, or cerebrovascular disease or high-risk diabetes with end organ damage. Previous users of an ARB/ACEi were eligible but were excluded if they were unable to discontinue use. Recruitment for the trial closed in 2003, and 25 620 patients were recruited. The primary outcome of the trial was a composite measure of cardiovascular-related death, MI, stroke, or hospitalization for heart failure.

### Study results

The primary outcome occurred in 1412 (16.5%) patients in the ramipril group and 1423 (16.7%) patients in the telmisartan group. For the primary composite outcome for telmisartan versus ramipril, the hazard ratio (HR) was 1.01 (95% CI, 0.94-1.09).^12^

## The emulation using observational data

### Data sources and study cohort

We aimed to emulate the ONTARGET trial’s primary objective by developing a propensity-score (PS)–weighted trial-eligible cohort in the UK Clinical Practice Research Datalink (CPRD) GOLD primary-care dataset. As of 2019, patients registered at practices currently contributing data to CPRD covered 4.32% of the UK population, and patients included were representative of the UK general population in terms of age, sex, and ethnicity.^16^^,^^17^ Primary-care data from the CPRD were linked to hospitalization data from the UK Hospital Episode Statistics and death registrations from the Office for National Statistics, with approximately 52% of patients in CPRD GOLD being eligible for linkage in 2019.^17^

Our study protocol has been published previously^18^; key components of the reference trial (ONTARGET), the hypothetical target trial, and emulation in CPRD GOLD are detailed in Table S1, with deviations detailed in Table S2. Steps for creating the study cohort are outlined below.

### Procedures

#### Exposures and outcomes

To maximize study power and generalizability, we compared outcomes between users of ARB and ACEi, rather than telmisartan and ramipril specifically. Outcomes were selected to replicate those in the ONTARGET trial.

Primary outcome: composite of cardiovascular death, MI, stroke, or hospital admission for congestive heart failure.
Secondary outcomes:

$ \circ $ Main secondary outcome: composite of cardiovascular death, MI, or stroke

$ \circ $ Individual components of primary outcome

$ \circ $ Death from noncardiovascular causes

$ \circ $ All-cause mortality
Further secondary and other outcomes: (separately) newly diagnosed congestive heart failure; revascularization procedures; decrease in glomerular filtration rate (GFR) or development of end-stage kidney disease (ESKD) (defined as a 50% reduction in estimated GFR (eGFR), the start of kidney replacement therapy, or development of an eGFR less than 15 mL/min/1.73 m^2^); development of ESKD (defined as the start of kidney replacement therapy or development of an eGFR less than 15 mL/min/1.73 m^2^); or microvascular complications of diabetes mellitus. GFR was calculated using the 2009 Chronic Kidney Disease Epidemiology Collaboration equation without reference to ethnicity.^19^
Safety outcomes: cough, angioedema, hyperkalemia (potassium concentration greater than 5.5 mmol/L), or ≥30% increase in serum creatinine concentration.

#### Treatment strategies

##### Step 1: create exposed periods

We selected patients who had ever been prescribed any dose of an ACEi and/or ARB from January 1, 2001, to July 31, 2019, and had been registered at an up-to-standard medical practice (meeting minimum data quality criteria^16^) for at least 12 months at the time of their first prescription (Figure S1). We defined “exposed periods” as all continuous courses of therapy, with a calculated prescription gap of more than 90 days being referred to as an “unexposed period.” We did not restrict the study cohort to new users; therefore, we started their follow-up at the start of any of the exposed periods for which they met trial criteria and were included in the cohort, thus emulating recruitment into the ONTARGET trial. Patients were not required to have a minimum length of exposure to be considered.

##### Step 2: create trial-eligible periods

Using Read diagnostic codes and *International Classification of Diseases, Tenth Revision* codes, we selected exposed periods that met the ONTARGET trial criteria. This resulted in a pool of trial-eligible exposed periods within individuals in the CPRD. Specific diagnostic codes used for cohort identification are available for download at https://doi.org/10.17037/DATA.00002112.

##### Step 3: balance across exposure groups

The original trial coordinator, the Population Health Research Institute, anonymized and provided access to the ONTARGET trial data, which included individual-level patient data for exposure and baseline covariates only. This was used to develop a trial-matched exposed cohort which was then used to inform an additional PS model to achieve balance across CPRD exposure groups (Figure S2). This trial-matched cohort was also used in a sensitivity analysis, which allowed us to examine whether trial results could be replicated when the CPRD cohort had a similar covariate distribution to the ONTARGET trial participants and when distribution differed. The latter enabled us to extend findings to underrepresented and excluded groups aided with a cohort with more diverse characteristics than the trial and more representative of the UK population receiving these medications in routine care.

In the main analysis, to ensure balance among CPRD groups used in analysis, we randomly selected 1 trial-eligible period per patient from the cohort of ARB and ACEi trial-eligible periods and generated PSs and obtained inverse probability weights,^20^ using a PS model for the probability of receiving an ACEi (Figure S3). Ensuring balance using PS weights instead of matching enabled us to maximize the number of participants included in the analysis. Patients could contribute to both ARB and ACEi exposed cohorts, but trial-eligible periods in the 2 exposure groups that had prescription start dates on the same day were excluded from both groups. Additional detail on achieving balance between groups is available in Appendix S1.

Variables included in the PS model were chosen based on a-priori knowledge of predictors of treatment with an ACEi and are displayed in Table S3. We considered comorbid conditions, medication history, demographic characteristics, and lifestyle factors. Because our cohort included prevalent users, we also included variables associated with switching treatments, such as time since first trial-eligible period and number of previous ARB/ACEi trial-eligible periods.^21^

### Statistical analysis

#### Benchmarking against the ONTARGET trial

Using an intention-to-treat approach for the main analysis, we compared cohorts using a Cox proportional hazards model weighted by PSs with robust SEs. The Cox model was additionally adjusted for any variables that demonstrated imbalance after PS weighting, using standardized differences with <0.1 as a cutoff.^22^ To replicate the trial per-protocol analysis, we also carried out an on-treatment analysis of ARB versus ACEi, additionally censoring at the date of discontinuation of the trial-eligible period—that is, calculated the end date of prescription when a subsequent prescription gap of more than 90 days occurred, when a patient switched treatment, or when a patient became a dual user plus 60 days (to allow for repeat prescriptions).

Because ONTARGET investigators reported relative risks for safety outcomes, we used a PS-weighted log-binomial model with robust SEs. Treatment cessation was defined as the end of an included trial-eligible exposed period (ie, a prescription gap of more than 90 days after the calculated prescription end date). The last safety event which occurred before treatment cessation was considered the reason for treatment cessation, and these results were compared with ONTARGET.

We emulated the subgroup analyses carried out in ONTARGET using a PS-weighted Cox proportional hazards model fitted with an interaction term for subgroup and treatment and used a Wald test to identify any effect modification. The subgroups studied were as in ONTARGET: sex, age (<65 years, 65-74 years, or ≥75 years), systolic blood pressure (SBP) (≤134 mm Hg, 135-150 mm Hg, or >150 mm Hg), diabetes, and cardiovascular disease at study entry. In addition, we included CKD status at baseline as a subgroup (CKD: eGFR <60 mL/min/1.73 m^2^).

#### Validation criteria

A priori, we defined replicability of the primary outcome of ONTARGET (HR = 1.01; 95% CI, 0.94-1.09) if the HR estimates from the PS-weighted analysis for ARB versus ACEi were between 0.90 and 1.12 and the 95% CI for the HR contained 1.0.^18^

### Extending findings to trial-underrepresented groups

Conditional on the validation criteria being met, we examined whether there was treatment heterogeneity among the underrepresented groups using interaction terms for sex, age, and CKD status. For CKD status, we repeated methods to create the PS-weighted cohort after removing the trial exclusion criterion of baseline serum creatinine concentration greater than 265 μmol/L.

### Sensitivity analyses

Reasons for potential differences in effect estimates in the reference trial and emulation in observational data that might lead to a false conclusion upon replication due to canceling out of biases were explored through design choices and sensitivity analyses (described in Table S4). To explore any benefits of using a PS-matched trial-eligible cohort, which ensured that patient characteristics were comparable to those of trial participants, as opposed to a PS-weighted trial-eligible cohort in which patients were more diverse, we 1:1 PS-matched ONTARGET participants to trial-eligible ACEi patients and then matched this trial-matched ACEi cohort to the closest trial-eligible ARB period and repeated the analyses (Figure S4).^18^ This is further detailed in Appendix S1.

To assess the impact of differential loss to follow-up in the reference trial and emulation, we reanalyzed excluded patients who were lost to follow-up in the first 12 months.

To examine the impact of including patients who may have only received 1 prescription for an ARB/ACEi, we started follow-up from 28 days after the start of the trial-eligible period, excluding patients if there were no prescriptions after 28 days.

We assessed the impact on the kidney outcomes of specifying sustained deterioration of kidney function. This required an eGFR less than 15 mL/min/1.73 m^2^ or a 50% reduction in eGFR on 2 occasions at least 3 months apart for decrease in eGFR or ESKD and development of ESKD outcomes.

To explore the bias introduced in a complete-case analysis for patients who had missing data for variables included in the PS model, we repeated analyses after imputing values for variables that could be assumed to missing at random using multiple imputation.^23^^,^^24^

As a post-hoc sensitivity analysis, we assessed the impact of changing between medications in CPRD for safety outcomes by restricting the cohort to patients’ first trial-eligible exposed period, and by excluding those with previous exposure to the alternative medication at any time before.

## Results

### Baseline characteristics

**Figure 1:**
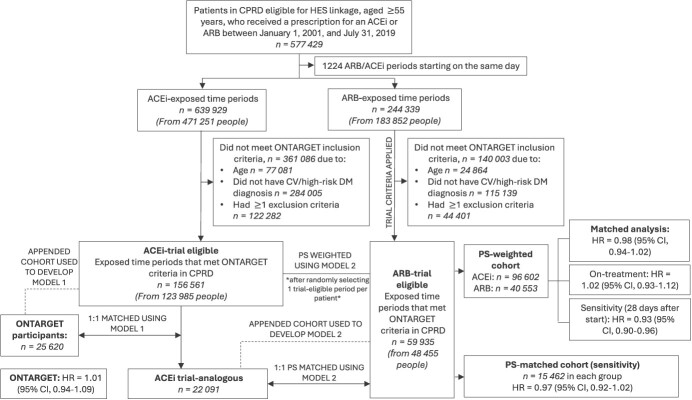
Study profile and steps to identify eligible patients included in analysis cohorts, Clinical Practice Research Datalink (CPRD), United Kingdom, 2001-2019. Model 1: Propensity score (PS) model for the probability of being included in the trial built using an appended cohort of ONTARGET trial participants and trial-eligible angiotensin-converting enzyme inhibitor (ACEi) patients. Model 2: PS model for the probability of receiving an ACEi built using the appended cohort of ACEi trial-analogous patients (obtained from 1:1 PS matching ONTARGET trial participants to the closest trial-eligible ACEi period using PS generated from model 1) appended to the angiotensin receptor blocker (ARB) trial-eligible patients. The PS-weighted analysis used inverse PS weights generated from conducting model 2 analyses on 1 randomly selected trial-eligible ARB period per patient and 1 randomly selected trial-eligible ACEi period per patient. The PS-matched analysis cohort used in sensitivity analysis was developed from 1:1 PS matching of trial-analogous ACEi patients to the closest trial-eligible ARB period using PSs generated from model 2. For the main analysis, follow-up began on the start date of the trial-eligible period and patients were censored at the earliest of the following dates: outcome, death, transferring out of the practice, last practice data collection, or 5.5 years from the start of the trial-eligible period, to reflect maximum follow-up of ONTARGET. In the on-treatment analysis, patients were additionally censored at the end of an eligible period, if they switched treatment, or if they started dual therapy plus 60 days. In the sensitivity analysis, follow-up started from 28 days after the beginning of the eligible period (to reflect the typical length of prescription), excluding patients if there were no prescriptions after 28 days. A total of 51 775 ACEi patients and 18 410 ARB patients were excluded for heart failure. Of the 1 randomly selected trial-eligible ARB and ACEi period per patient, 27 383 ACEi trial-eligible patients and 7902 ARB trial-eligible patients were excluded from the analysis due to having missing values for variables included in the PS model. Actual numbers of patients included in the PS-weighted cohort were 96 602 and 40 553 in the ACEi and ARB groups, respectively. The weighted distributions of patients in the analysis cohort were 145 257.7 and 135 592.3 in the ACEi and ARB groups, respectively. CV, cardiovascular; DM, diabetes mellitus; HES, Hospital Episode Statistics; HR, hazard ratio.

After PS weighting, 96 602 ACEi-prescribed patients and 40 553 ARB-prescribed patients were included in the comparison of ARB users versus ACEi users, with weighted distributions of 145 257.7 and 135 592.3 in the ACEi and ARB groups, respectively (Figure 1). In the ARB group, the median PS weight was 2.42, with minimum of 1.02 and a maximum of 36.09. In the ACE inhibitor group, the median PS weight was 1.21, with a minimum of 1.02 and a maximum of 86.08.

Mean ages were similar across exposure groups (71 years), slightly older than in ONTARGET (66 years). There was a higher proportion of females across exposure groups (approximately 51%) than in ONTARGET (27%) (Table 1). Balance before and after weighting is shown in Table S5. Imbalance remained for several time-related variables—time since first trial-eligible period, calendar year of trial-eligible period, and number of prior ARB trial-eligible periods—so results were adjusted for these variables.

**Table 1:** Baseline characteristics of Clinical Practice Research Datalink trial-eligible patients after application of trial criteria included in PS-weighted analysis as compared with ONTARGET, United Kingdom, 2001-2019

	**CPRD medication**				**ONTARGET** ^**a**^ **(*n* = 25 620)**	
**Characteristic**	**ACEI (*n* = 96 602)**		**ARB (*n* = 40 553)**			
	**Mean (SD)**	**No. (%)**	**Mean (SD)**	**No. (%)**	**Mean (SD)**	**No. (%)**
Age, y	70.8 (9.0)		71.2 (8.7)		66.4 (7.2)	
Blood pressure, mm Hg						
Systolic	147.4 (20.7)		148.1 (20.7)		141.8 (17.4)	
Diastolic	80.1 (10.7)		79.7 (10.5)		82.1 (10.4)	
Body mass index^b^	28.3 (5.3)		28.8 (5.4)		28.2 (4.7)	
Cholesterol, mmol/L	4.8 (1.2)		4.7 (1.1)		4.9 (1.2)	
Triglycerides, mmol/L	1.7 (1.0)		1.6 (0.9)		1.7 (1.1)	
Glucose, mmol/L	6.5 (2.5)		6.4 (2.4)		6.7 (2.6)	
Creatinine, μmol/L	93.9 (25.0)		94.3 (26.9)		94.2 (24.4)	
Potassium, mmol/L	4.4 (0.5)		4.4 (0.5)		4.4 (0.4)	
Female sex		45 508 (47.1)		22 690 (56.0)		6831 (26.7)
Ethnic group						
Black		1280 (1.3)		736 (1.8)		629 (2.5)
Other		1134 (1.2)		607 (1.5)		4901 (19.1)
South Asian		3026 (3.1)		1799 (4.4)		1375 (5.4)
Unknown						7 (<0.1)
White		91 162 (94.4)		37 411 (92.3)		18 708 (73.0)
Clinical history						
Coronary artery disease^c^		68 009 (70.4)		28 202 (69.5)		19 102 (74.6)
Myocardial infarction		21 997 (22.8)		7301 (18.0)		12 549 (49.0)
Angina pectoris		31 595 (32.7)		13 205 (32.6)		11 505 (44.9)
Cerebrovascular disease^d^		8695 (9.0)		3140 (7.7)		5342 (20.9)
Peripheral artery disease^e^		9999 (10.4)		4078 (10.1)		3468 (13.5)
Diabetes		43 751 (45.3)		20 003 (49.3)		9612 (37.5)
High-risk diabetes^f^		30 736 (31.8)		14 757 (36.4)		7151 (27.9)
Previous surgical procedures						
CABG		6747 (7.0)		2912 (7.2)		5675 (22.2)
PTCA		10 055 (10.4)		3823 (9.4)		7437 (29.0)
Chronic kidney disease^g^		27 608 (28.6)		13 246 (32.7)		5470 (21.4)
Smoking status						
Nonsmoker		34 503 (35.7)		16 470 (40.6)		9088 (35.5)
Current smoker		12 921 (13.4)		3552 (8.8)		3225 (12.6)
Past smoker		49 178 (50.9)		20 531 (50.6)		13 276 (51.8)
Unknown						31 (0.1)
Alcohol consumption status						
Drinker		76 756 (79.5)		31 840 (78.5)		10 345 (40.4)
Nondrinker		19 846 (20.5)		8713 (21.5)		15 261 (59.6)
Unknown						14 (<0.1)
Medication use^h^						
ACE inhibitor		78 287 (81.0)		4659 (11.5)		14 750 (57.6)
Alpha blocker		6892 (7.1)		3202 (7.9)		1095 (4.3)
Oral anticoagulant agent		4613 (4.8)		1282 (3.2)		1939 (7.6)
Antiplatelet agent^i^		12 334 (12.8)		2482 (6.1)		2824 (11.0)
Angiotensin receptor blocker		440 (0.5)		34 579 (85.3)		2213 (8.6)
Aspirin		44 011 (45.6)		9325 (23.0)		19 403 (75.7)
Beta blocker		34 178 (35.4)		6756 (16.7)		14 583 (56.9)
Calcium-channel blocker		28 820 (29.8)		8515 (21.0)		8472 (33.1)
Digoxin		2533 (2.6)		600 (1.5)		865 (3.4)
Diuretic		32 002 (33.1)		8838 (21.8)		7164 (28.0)
Diabetic treatment		20 060 (20.8)		4910 (12.1)		8056 (31.4)
Nitrate		14 862 (15.4)		3172 (7.8)		7523 (29.4)
Statin		52 925 (54.8)		11 474 (28.3)		15 783 (61.6)

Abbreviations: ACE, angiotensin-converting enzyme; CABG, coronary artery bypass graft; ONTARGET, Ongoing Telmisartan Alone and in Combination with Ramipril Global Endpoint Trial; PTCA, percutaneous transient coronary angioplasty.

^a^One third of ONTARGET participants received both ramipril and telmisartan.

^b^Weight (kg)/height (m)^2^.

^c^Includes diagnosis of myocardial infarction at least 2 days prior, angina at least 30 days prior, angioplasty at least 30 days prior, or CABG at least 4 years prior.

^d^Includes diagnosis of stroke or transient ischemic attack.

^e^Includes diagnosis of limb bypass surgery, limb/foot amputation, or intermittent claudication.

^f^Includes diabetes mellitus with retinopathy, neuropathy, chronic kidney disease, proteinuria, or another complication.

^g^Estimated glomerular filtration rate less than 60 mL/min/1.73 m^2^.

^h^Within 3 months prior to the eligible start date.

^i^Clopidogrel/ticlopidine.

### Follow-up and adherence

In the PS-weighted trial-eligible cohort, a total of 82 121 patients (ACEi: *n* = 58553; ARB: *n* = 23568) were followed until they experienced an event or completed 5.5 years of follow-up (maximum follow-up in the ONTARGET trial). A total of 10 046 patients were censored at death, 32 034 patients were censored at the date on which the practice last contributed data to the CPRD, and 13 124 patients transferred out of the practice. After 1 year, among patients in the ARB group, 2.6% had switched to an ACEi, and among patients in the ACEi group, 11% had switched to an ARB. Adherence was lower in the CPRD, with 70% of ACEi patients still on ACEi treatment after 1 year and 78% of ARB patients still on ARB treatment after 1 year, as compared with ONTARGET, where 92% of ramipril patients were taking an ACEi and 94% of telmisartan patients taking an ARB after 1 year.^12^ However, only small differences were observed between ARB and ACEi exposure groups in the CPRD (Table S6).

### Benchmarking results

#### Primary outcomes and validation

**Figure 2:**
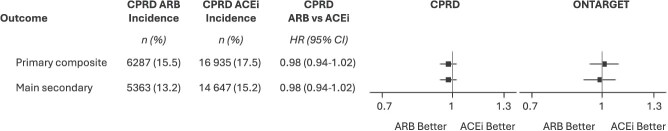
Hazard ratios (HRs) from PS-weighted and adjusted analysis of angiotensin receptor blocker (ARB) use versus angiotensin-converting enzyme inhibitor (ACEi) use for the primary composite outcome (cardiovascular death, myocardial infarction, stroke, or hospitalization for heart failure) and the main secondary outcome (cardiovascular death, myocardial infarction, or stroke) as compared with telmisartan versus ramipril in the ONTARGET Trial (Ongoing Telmisartan Alone and in Combination with Ramipril Global Endpoint Trial), Clinical Practice Research Datalink (CPRD), United Kingdom, 2001-2019. *n* = number of events. Bars, 95% CIs.

In the PS-weighted trial-eligible cohort, the primary composite outcome occurred among 6287 (16%) patients in the ARB group and among 16 935 (18%) patients in the ACEi group (median follow-up, 4.7 years), for event rates of 4.2 per 100 person-years and 4.4 per 100 person-years, respectively. In ONTARGET, the numbers of events were 1423 (17%) and 1412 (17%) in the telmisartan and ramipril treatment groups, respectively, over a median follow-up period of 4.7 years. Comparing ARB users with ACEi users in the trial-eligible cohorts, the risks of the primary outcome were similar (HR 0.98 [95% CI, 0.94-1.02] in the PS-weighted, adjusted analysis). This was comparable to the ONTARGET primary outcome (HR = 1.01; 95% CI, 0.94-1.09) and met the prespecified validation criteria of trial replicability (Table 2 and Figure 2). The Kaplan–Meier plot showed a lower risk among ARB users than among ACEi users at 1, 2, 3, 4, and 5 years of follow-up (Figure S5). This differed from the ONTARGET results, which showed a consistent risk up to 1.5 years and then a lower risk among ACEi users. Results of the on-treatment analysis showed that ARBs were associated with a decreased risk of the primary composite outcome (HR = 0.90; 95% CI, 0.86-0.94) for ARB versus ACEi.

**Table 2:** Numbers of events for the primary outcome, its components, and death from any cause in a propensity-score–weighted analysis of ARB versus ACEi using CPRD data, United Kingdom, 2001-2019

**Outcome** ^**a**^	**CPRD medication,** ^**b**^ **no. (%)**		**Medication comparison, hazard ratio (95% CI)**	
	**ACEI (*n* = 96 602)**	**ARB (*n* = 40 553)**	**ARB vs ACEI** **(CPRD** ^**c**^ **; *n* = 137 155)**	**Telmisartan vs ramipril** **(ONTARGET** ^**d**^ **; *n* = 17 118)**
Primary composite outcome: death from CV causes, MI, stroke, or hospitalization for heart failure	16 935 (17.5)	6287 (15.5)	0.98 (0.94-1.02)	1.01 (0.94-1.09)
Main secondary outcome: death from CV causes, MI, or stroke	5363 (13.2)	14 647 (15.2)	0.98 (0.94-1.02)	0.99 (0.91-1.07)
MI	11 617 (12.0)	4090 (10.1)	0.97 (0.92-1.01)	1.07 (0.94-1.22)
Stroke	3768 (3.9)	1573 (3.9)	1.04 (0.97-1.12)	0.91 (0.79-1.05)
Hospitalization for heart failure	4028 (4.2)	1570 (3.9)	0.97 (0.90-1.05)	1.12 (0.97-1.29)
Death from CV causes	5194 (5.4)	1825 (4.5)	0.96 (0.90-1.03)	1.00 (0.89-1.12)
Death from non-CV causes	6984 (7.2)	2649 (6.5)	0.97 (0.92-1.02)	0.96 (0.83-1.10)
Death from any cause	12 178 (12.6)	4474 (11.0)	0.97 (0.93-1.01)	0.98 (0.90-1.07)

Abbreviations: ACEi, angiotensin-converting enzyme inhibitors; ARB, angiotensin receptor blockers; CPRD, Clinical Practice Research Datalink; CV, cardiovascular; MI, myocardial infarction; ONTARGET, Ongoing Telmisartan Alone and in Combination with Ramipril Global Endpoint Trial.

^a^MI and stroke included both fatal and nonfatal events.

^b^A total of 55 015 (57.0%) ACEi patients included received ramipril as the first prescription for the included trial-eligible exposed period, and 1495 (3.7%) ARB patients included received telmisartan as the first prescription for the included trial-eligible exposed period.

^c^The CPRD-weighted analysis included 1 randomly selected trial-eligible period per patient. The analysis was a PS-weighted analysis with robust SEs. Results were adjusted for time since first eligible period, number of prior ARB periods, and calendar year.

^d^ONTARGET results are from published findings.^12^

#### Secondary and other outcomes

Results were consistent with ONTARGET for the main secondary composite outcome of cardiovascular death, MI, or stroke (Figure 2) and all other secondary outcomes, including development of ESKD (HR = 1.06; 95% CI, 0.95-1.19) (Table S7). However, within the CPRD trial-eligible cohort, the risk of the composite of loss of GFR or ESKD was higher for ARB users than for ACEi users (HR = 1.11; 95% CI, 1.04-1.19), where ONTARGET observed similar treatment effects (Table 2 and Table S7).

#### Safety outcomes

In analyses of safety outcomes as reasons for treatment cessation, cough was more common in ARB users than in ACEi users (RR = 1.29; 95% CI, 1.16-1.43) and angioedema rates were similar between groups, both in contrast with ONTARGET findings of reduced risk of cough and angioedema with ARB versus ACEi; however, the number of events in our analysis was low, and our assessment was based on timing, whereas the ONTARGET reason for discontinuation was prospectively documented. Hyperkalemia and a ≥30% increase in serum creatinine concentration were also more common in ARB users than in ACEi users (RR = 1.12 [95% CI, 1.06-1.18] and RR = 1.38 [95% CI, 1.34-1.43], respectively) (Table S8).

#### Subgroup analysis

**Figure 3:**
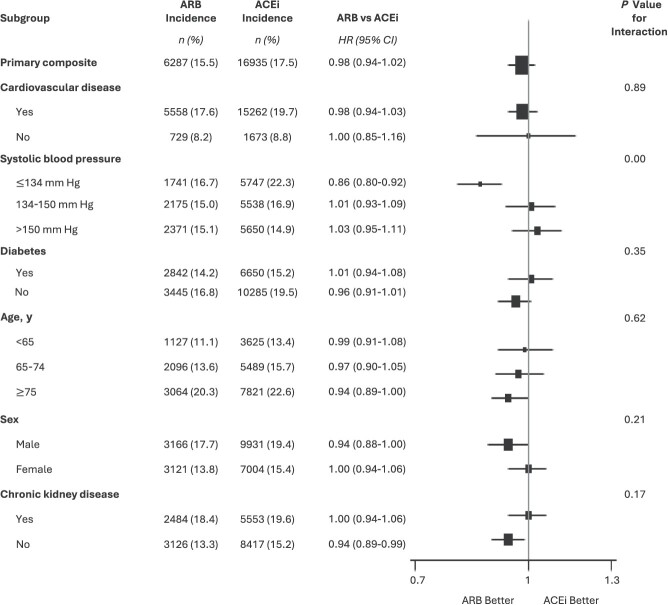
Hazard ratios (HRs) in prespecified subgroups that were studied in ONTARGET (Ongoing Telmisartan Alone and in Combination with Ramipril Global Endpoint Trial), including underrepresented groups of females and persons aged ≥75 years, along with those with chronic kidney disease (not analyzed in ONTARGET and underrepresented), for comparison between angiotensin receptor blocker (ARB) use and angiotensin-converting enzyme inhibitor (ACEi) use in analysis of the primary composite outcome (cardiovascular death, myocardial infarction, stroke, or hospitalization for heart failure), Clinical Practice Research Datalink (CPRD), United Kingdom, 2001-2019. *n* = number of events. The *P* value reflects the significance of the test for interaction between the treatment and each subgroup. Cardiovascular disease comprised patients with coronary artery disease, peripheral artery disease, or cerebrovascular disease. Chronic kidney disease was defined as an estimated glomerular filtration rate less than 60 mL/min/1.73 m^2^. Bars, 95% CIs.

Results for the primary outcome for ARB versus ACEi, stratified within the same subgroups as ONTARGET, are shown in Figure 3. We observed evidence of effect modification by baseline SBP (*P* <.01), with a lower risk among ARB users compared with ACEi users in those with baseline SBP *≤*134 mm Hg (HR = 0.86; 95% CI, 0.80-0.92). All other subgroups studied showed no strong evidence of treatment heterogeneity between groups, which was consistent with the findings in ONTARGET.

### Underrepresented groups

For ARB versus ACEi for the primary composite outcome, there was no evidence to suggest treatment heterogeneity by sex (males: HR = 0.94 [95% CI, 0.88-1.00]; females: HR = 1.00 [95% CI, 0.94-1.06]; *P* =.21), by age group (<65 years: HR = 0.99 [95% CI, 0.91-1.08]; 65-75 years: HR = 0.97 [95% CI, 0.90-1.05]; ≥75 years: HR = 0.94 [95% CI, 0.89-1.00]; *P* =.62), or by CKD status (no CKD: HR = 0.94 [95% CI, 0.89-0.99]; CKD: HR = 1.00 [95% CI, 0.94-1.06]; *P* =.17), with all groups showing equivalent treatment effects of ARB and ACEi use. Among the trial-underrepresented groups of females, persons aged ≥75 years, and those with CKD, treatment effects for the primary composite outcome were consistent with ONTARGET (Figure 3).

For most secondary outcomes, treatment effects were similar among males and females. However, there was some evidence of treatment heterogeneity for the outcomes of cardiovascular-related death (males: HR = 1.02 [95% CI, 0.93-1.12]; females: HR = 0.88 [95% CI, 0.80-0.97]; *P =.*03), all-cause mortality (males: HR = 1.00 [95% CI, 0.94-1.06]; females: HR = 0.91 [95% CI, 0.85-0.97]; *P =.*04), and revascularization procedures (males: HR = 0.96 [95% CI, 0.91-1.01]; females: HR = 1.06 [95% CI, 0.98-1.15]; *P =.*03). Treatment effects were similar among ARB and ACEi users for men, but among females ARB use was associated with lower risks of cardiovascular-related death and all-cause mortality compared with ACEi use (Figure S6).

Similarly, by age group there was no evidence of treatment heterogeneity for most secondary outcomes. However, treatment effects differed for the outcomes of revascularization procedures (<65 years: HR = 0.91 [95% CI, 0.84-0.98]; 65-74 years: HR = 1.04 [95% CI, 0.97-1.12]; ≥75 years: HR = 1.03 [95% CI, 0.95-1.12]; *P =.*02) and decrease in GFR or ESKD (<65 years: HR = 1.42 [95% CI, 1.22-1.66]; 65-74 years: HR = 1.09 [95% CI, 0.97-1.22]; ≥75 years: HR = 1.00 [95% CI, 0.91-1.10]; *P* < 0.01). ARB and ACEi had similar treatment effects among users aged ≥65 years, but among users aged <65 years, ARB use was associated with a lower risk of revascularization procedures and a higher risk of decrease in GFR or ESKD, but event numbers were low (Figure S7).

For CKD, evidence of treatment heterogeneity was observed for MI (no CKD: HR = 0.92 [95% CI, 0.86-0.99]; CKD: HR = 1.05 [95% CI, 0.97-1.14]; *P =.*02), newly diagnosed heart failure (no CKD: HR = 0.91 [95% CI, 0.84-0.98]; CKD: HR = 1.02 [95% CI, 0.95-1.10]; *P =.*03), and revascularization procedures (no CKD: HR = 0.95 [95% CI, 0.89-1.00]; CKD: HR = 1.08 [95% CI, 0.99-1.17]; *P* =.01). For these outcomes, treatment effectiveness was similar among ARB and ACEi users with CKD at baseline, but ARB use was associated with a lower risk among those without CKD at baseline (Figure S8).

### Sensitivity analyses

The PS-matched trial-eligible cohort, with a similar covariate distribution as the ONTARGET trial participants, included 15 462 patients in the ARB and ACEi exposure groups, respectively. The distribution after PS matching is displayed in Figures S9 and S10 and Table S9. Analysis of the PS-matched trial-eligible cohort for ARB versus ACEi gave results similar to those of the PS-weighted trial-eligible cohort for the primary outcome (HR = 0.97 [95% CI, 0.92-1.02]; numbers of events—ARB: *n* = 2453 events [16%], ACEi: *n* = 2539 events [16%]) (Table 2 and Table S10). For all other outcomes, results had HRs close to 1.0 and 95% CIs containing 1.0 (Table S10).

Excluding patients who were lost to follow-up in the first 12 months gave consistent results (HR = 0.96; 95% CI, 0.93-1.00).

The risk of the primary outcome was lower among ARB users when follow-up was started from 28 days after the beginning of the trial-eligible period (HR = 0.93 [95% CI, 0.90-0.96]; numbers of events—ARB: *n* = 5966 [15%], ACEi: *n* = 16 051 [16.8%]).

Specifying sustained deterioration of kidney function for loss of GFR or ESKD had no effect on results. However, among ARB users, the risk of development of ESKD was increased (HR = 1.16 [95% CI, 1.02-1.32]; numbers of events—ARB: *n* = 626 [1.7%], ACEi: *n* = 1016 [1.2%]).

After imputation of missing blood pressure values, 159 651 patients were included in analysis. For the primary composite outcome, for ARB use versus ACEi use, the PS-weighted Cox proportional hazards model gave an HR of 0.95 [95% CI, 0.92-0.98].

Restriction of the analysis to new users with no previous exposure to the opposite medication for safety outcomes showed a lower risk of cough and angioedema as the reason for treatment cessation for ARB use versus ACEi use, which was consistent with the ONTARGET findings (Table S11).

## Discussion

We emulated the ONTARGET randomized trial using a large dataset with routinely collected health-care data. By applying the trial criteria and creating a PS-weighted trial-eligible cohort with balanced characteristics in each treatment arm, we showed similar risks among ARB and ACEi users for the composite outcome of cardiovascular death, MI, stroke, or hospital admission for congestive heart failure, as well as further secondary outcomes. We attempted to emulate the ONTARGET per-protocol analysis using an on-treatment approach where we obtained inconsistent results. It was suspected that this was due to increased medication channeling in the early years, which introduced bias into results and differences in groups for measured and unmeasured confounders. This was assessed in a post-hoc analysis where we stratified by calendar year of the start of the trial eligible period; and when restricting the cohort to eligible periods between 2010 and 2019 (ie, after the ONTARGET findings were published), we observed results consistent with those of the main analysis (HR = 1.06; 95% CI, 0.96-1.17). Marked similarity between ONTARGET and our observational study was found in subgroup analysis, with ARB users with the lowest baseline SBP being at lower risk of the primary composite outcome compared with ACEi users. This could indicate that persons with less severe hypertension may be given an ARB, which is commonly seen with new medications prescribed to healthier patients, introducing some bias.

We subsequently extended analysis to females, persons aged ≥75 years, and patients with CKD (all underrepresented in ONTARGET), where we demonstrated consistency of treatment effects for most outcomes but saw some evidence to suggest that ARB use was associated with a lower risk of death-related outcomes among females.

### Comparison with other studies

Our findings of similar effectiveness of ARB versus ACEi by sex and age for a composite cardiovascular outcome were consistent with previous comparative effectiveness studies.^25^^,^^26^ In line with the findings from a large Taiwanese cohort study,^27^ we demonstrated little difference between ARB use and ACEi use in risk of kidney outcomes among persons with and without CKD.

In one recent ONTARGET replication study using US insurance claims data, Fralick et al^6^ performed a PS-matched analysis of telmisartan versus ramipril and found an HR of 0.99 (95% CI, 0.85-1.14) for the primary outcome. The sample was small (9930 patients) and, unlike the trial, included new users only.

In contrast to other naive observational studies that have shown a decreased risk among ARB users,^28^^-^^30^ we observed equal treatment effectiveness of ARB and ACEi. This implies that using trial emulation techniques and PS weighting to obtain balance among exposure groups can adequately address confounding and bias and lead to results comparable to those of the reference trial.

### Strengths and limitations

We were able to demonstrate that both a PS-weighting approach and a PS-matching approach yielded results equivalent to those of ONTARGET, providing evidence to support the use of a weighted approach in future trial replication studies which aim to explore whether trial results extend to underrepresented groups. Having replicated the ONTARGET results, the increased sample size and diverse population in the PS-weighted trial-eligible cohort allowed us to extend our analyses to trial-underrepresented groups. This included people with CKD, where evidence from observational studies is limited. In this group, we observed similar treatment effectiveness among ARB and ACEi users for the primary outcome and all other outcomes, including the outcomes of loss of GFR or ESKD and development of ESKD.

Despite overall similarity between ARB and ACEi users for most outcomes, we noted some discrepancies with ONTARGET. In the ONTARGET trial, ARB and ACEi users had comparable risks of kidney-related outcomes. In contrast, we found ARBs to be associated with a moderately greater risk of decrease in GFR or ESKD compared with users of ACEi. This may reflect testing of multiple outcomes, low numbers of outcomes in some strata, or residual confounding by indication.

The use of robust SEs with PS weighting may have led to overestimation of the SEs, which may have led to wider CIs.^31^

When dealing with comparisons between a new medication and a historical medication, careful considerations need to be given to handling treatment switchers and appropriately accounting for time trends in prescribing. We sought to account for such variables, including them as terms in our PS model, but it is not possible to exclude this as a source of residual confounding or bias. The ONTARGET trial included a 3-week run-in period, and prior to randomization 3399 patients were excluded. By design of the run-in, tolerance and adherence to ramipril use was assessed more thoroughly than that of telmisartan use. Subsequently, exclusion of intolerant patients prior to randomization may have led to bias in results in favor of ramipril.^32^ We were unable to identify medication-intolerant patients prior to study entry in our emulation, which used routine data. This indicates a difference in design between our emulation and the ONTARGET trial. However, we are confident that this difference and any potential bias in the reference trial caused by the run-in was likely to have been minimal due to the consistent results we observed between our emulation and the reference trial. Despite this, for some outcomes we observed a decrease in risk associated with ARB use whencompared with ACEi use, which indicates that although the bias may have been minimal, it was not eliminated. Starting follow-up from 28 days after the beginning of the trial-eligible period to assess the impact of including patients who received only 1 prescription led to a lower risk of the primary outcome among ARB users. This could indicate that our main analysis might have included patients who had briefly switched to an ARB before switching back to their original treatment. Therefore, the event captured may have been incorrectly attributed to ARB use’s indicating equivalent treatment effects when in fact ARBs were associated with fewer events. This indicates that some bias may still remain in relation to treatment switchers.

Discrepancy of safety outcomes is likely due to the close monitoring of adverse events in a trial setting as compared with routine clinical care. Events such as cough are likely to be underreported in routine data. In addition to this, some confounding by indication may be present, particularly for patients with a history of cough or angioedema who may have been switched from an ACEi to an ARB. This was demonstrated in our sensitivity analysis restricting the cohort to nonswitchers, where we obtained results much closer to those of the ONTARGET trial. However, since ARB users who have not previously been exposed to an ACEi are likely to be healthier and less likely to experience cough, due to the known risk of cough among ACEi users, we cannot be sure that restricting to nonswitchers does not introduce further bias. Furthermore, to replicate the safety analysis presented in the trial, we used a log-binomial model to estimate relative risks. Therefore, we assumed right-censoring to have minimal effect.

We used PS methods to achieve balance across CPRD exposure groups and assumed that the variables in the PS model sufficiently accounted for measured confounding. For efficiency to achieve balance across CPRD exposure groups, the same PS model was used in the main weighted analysis and the PS-matched sensitivity analysis. This model was developed using appended trial-analogous ACEi and trial-eligible ARB patients. Therefore, development of the PS-weighted analysis cohort was not independent of the reference trial. Due to lack of randomization in observational studies, we assumed no unmeasured confounding conditional on the measured confounders included in the PS model. We also assumed consistency and no interference and examined the PS distribution to evaluate the positivity assumption. Some patients were excluded due to missing data for variables included in the PS model. Less than 5% of values were missing for most variables included in the PS model. However, approximately 14% of patients had missing blood pressure data at baseline and were excluded. Therefore, our analysis was conditional on nonmissing values for variables included in the PS model, and we assumed that the 14% of patients with missing blood pressure values were missing data at random and that this had no effect on results. We assessed the implication of these assumptions using multiple imputation to impute missing blood pressure values and observed a point estimate consistent with the main results but CIs which suggested that ARBs were associated with a decrease in risk of the primary composite outcome. This could be due to the increase in sample size and hence narrower CIs or could suggest bias in our main analysis, which included complete cases only.

In addition to unmeasured confounding and missing data, it is possible that other factors associated with study design and analysis could have biased our results. Each factor could potentially bias results in different directions and subsequently balance out, leading to a false conclusion on trial replicability. We sought to address these factors through design choices and sensitivity analyses (described in Table S4).

For example, differences between the emulation study and ONTARGET could be due to the treatment effect varying across groups that are unequally represented in the emulation and ONTARGET. Differences in study populations leading to differences in effect estimates may occur if not all of the trial eligibility can be implemented in the observational data. Some criteria were omitted, such as planned cardiac surgery due to misclassification. However, the number of criteria we were not able to apply was small.

The incidence of MI in the CPRD was higher than in ONTARGET, which could have led to a difference in effect estimates.

We explored adherence after treatment by calculating the proportion of patients who were still receiving treatment at 1, 2, 3, 4, and 5.5 years, as was done in the trial. Adherence in the CPRD differed from that in ONTARGET, with slightly worse adherence among ACEi users compared with ARB users, which was likely due to more patients switching to the newer medication. This is a limitation of studying a new drug versus an old drug in observational data, and in combination with residual confounding it could explain why risk was lower among ARB users in the Kaplan–Meier plot compared with what was observed in ONTARGET.

Finally, some differences in estimates may be caused by the use of different treatment strategies and causal contrasts. Unlike ONTARGET, to increase statistical power we studied the effects of medication classes as opposed to specific medications. We also attempted to emulate the reference trial per-protocol effect by additionally censoring patients at the end of a trial-eligible period—that is, when it is not clear whether the patient is still receiving assigned treatment, or when the patient switched treatment or started dual therapy—and estimated the on-treatment effect. It is suggested that the per-protocol effect should be reestimated in the ONTARGET trial and emulation adjusting for pre- and postbaseline information associated with adherence.^11^^,^^33^^,^^34^ However, since we did not have access to the outcome data from ONTARGET, we were unable to estimate this in the reference trial. Due to the nature of the data source, we also were unable to determine whether patients discontinued the medication for clinical reasons; therefore, informative censoring may have affected results. However, the number of patients who were additionally censored for discontinuation, switching, or dual use was small (3.6%).

### Conclusion

In this emulation of the ONTARGET randomized trial using routinely collected health-care data, we closely replicated the primary and secondary outcomes and were able to demonstrate the generalizability of ONTARGET trial results to a cohort representative of patients receiving prescriptions for ACEi or ARB in UK primary care. Subsequently, we were able to provide evidence that ONTARGET trial results extend to trial-underrepresented subgroups where evidence is limited, including females, persons aged ≥75 years, and patients with CKD. Benchmarking findings from observational studies against those of a reference trial can add confidence to findings when using routinely collected data to investigate the generalizability of trial findings to wider populations.

## Supplementary Material

Web_Material_kwae137

## Data Availability

No additional data are available.
