# Supplementary material for: Cardiorenal effects of angiotensin-converting enzyme inhibitors and angiotensin receptor blockers among people underrepresented in trials: analysis of routinely collected data with emulation of a reference trial (ONTARGET)
Source: Am J Epidemiol. 2024 Jun 18;193(12):1785–95. doi: 10.1093/aje/kwae137 (PMC11637514; doi:10.1093/aje/kwae137)
Supplement: Web_Material_kwae137 [file web_material_kwae137.pdf]

SUPPLEMENTARY MATERIAL

Cardiorenal effects of Angiotensin-converting enzyme inhibitors and Angiotensin receptor blockers in people underrepresented in trials: analysis of routinely collected data with emulation of a reference trial (ONTARGET)

Table of Contents

Item	Description	Page No.
Appendix 1	Additional details on Step 3: Balance across exposure groups	3
Table S1	Key design aspects of the reference trial (ONTARGET), the target trial and deviations from protocol with emulation in CPRD GOLD data.	6
Table S2	Deviations from protocol	9
Table S3	List of variables considered and included in propensity-score model used to achieve balance across exposure groups	11
Table S4	Explanation for potential differences in estimates from ONTARGET and the emulation in CPRD after mapping protocol components	13
Table S5	Assessment of balance of variables included in propensity-score model for ARB vs ACEi analysis before and after weighting	15
Table S6	Medication adherence to assigned exposure group	18
Table S7	Secondary and other outcomes after propensity-score—weighted analysis using CPRD data	19
Table S8	Reason for treatment cessation using trial criteria and propensity-score—weighting for ARB vs ACEi compared to ONTARGET	20
Table S9	Assessment of balance of variables included in propensity-score model for ARB vs ACEi sensitivity analysis after matching compared to ONTARGET	21
Table S10	Number of events in the primary outcome, its components, and death from any cause for ARB vs ACEi using a propensity-score—matched analysis of patients in CPRD (sensitivity analysis)	24
Table S11	Safety outcomes assessed among non-switchers using trial criteria and propensity-score—weighting for ARB vs ACEi (sensitivity analysis)	25
Figure S1	Study diagram	26
Figure S2	Steps to develop propensity score models used to achieve balance across CPRD exposure groups	26
Figure S3	Steps to achieve balance across exposure groups in main analysis using propensity-score—weighting	27
Figure S4	Steps to achieve balance across exposure groups in sensitivity analysis using propensity-score—matching	28
Figure S5	Kaplan-Meier curves for the Primary composite outcome for ARB and ACEi users.	28
Figure S6	Treatment heterogeneity by sex for all outcomes for comparison of ARB vs ACEi.	29
Figure S7	Treatment heterogeneity by age group for comparison of ARB vs ACEi.	30
Figure S8	Treatment heterogeneity by CKD status for all outcomes for comparison of ARB vs ACEi.	31

Figure S9	Kernel density plots before and after matching trial-matched ACEi patients to trial-eligible ARB patients	32
Figure S10	Kernel density plots before and after matching ONTARGET participants to trial-eligible ACEi patients	32

Appendix S1

Additional details on Step 3: Balance across exposure groups

As we had access to individual patient level data from the reference trial (ONTARGET) we aimed to carry out analysis on two cohorts and examine if this provided any benefit to obtaining results consistent with the reference trial. The analysis cohorts used were:

1) Main analysis: propensity-score—weighted trial-eligible groups which resulted in covariate distribution more diverse than the trial and representative of patients receiving these medications in routine care

2) Sensitivity analysis: propensity-score—matched trial-matched ACEi cohort to trial-eligible ARB cohort which resulted in covariate distribution similar to the reference trial

For efficiency the same propensity score model used to achieve balance across CPRD exposure groups was used for both analysis cohorts 1) and 2) above. The propensity model was developed for the probability of receiving an ACEi using an appended cohort of trial-matched ACEi patients and trial-eligible ARB periods. This is displayed graphically in Figure S2 (denoted as PS model 2).

The propensity-score—weighted analysis cohort, which was the main analysis presented in the paper, allowed us to extend findings to underrepresented groups. To develop this cohort we applied the propensity score model for the probability of receiving an ACEi (displayed as model 2 in Figure S2) to a cohort of one-randomly selected trial eligible period per patient from the trial-eligible ARB and ACEi exposure groups and generated inverse probability weights from propensity scores (Figure S3). Using the trial-eligible cohort enabled characteristics to be more diverse than the trial. We selected one random period as opposed to the first period to avoid the possibility of biasing results towards new users, since the reference trial included prevalent users.

For the sensitivity analysis, we 1:1 matched trial-analogous (trial-matched) ACEi patients to one-randomly selected trial-eligible period per patient from the trial-eligible ARB exposure group using propensity scores generated from the PS model used to achieve balance across CPRD exposure groups for the probability of receiving an ACEi (PS model 2 in Figure S2), with the restriction that only one ARB trial-eligible exposed period per patient could be matched (Figure S4). This analysis allowed us to explore if there was an added benefit of using a trial-analogous cohort with covariate distribution consistent with the reference trial. We used standardised differences (<0.1) and kernel density plots to assess the quality of matches which are displayed in Figure S10 and Supplementary Table S9.

For efficiency, we used the same model to balance exposure groups in CPRD (PS model 2 in Figure S2) for the propensity-score—weighted and propensity-score—matched sensitivity analysis. To develop this model we appended the trial-analogous ACEi cohort with the trial-eligible ARB cohort and generated a propensity score model for the probability of receiving an ACEi. Therefore, we acknowledge that despite the propensity-score—weighted analysis cohort not directly consisting of trial-analogous patients as the developed PS model was applied to trial-eligible patients, the ONTARGET data was used to inform this cohort. To develop this trial-anaologous ACEi cohort, which was used to ensure covariate distribution was consistent with the ONTARGET, ONTARGET trial data were combined with the CPRD cohort of trial-eligible ACEi exposed periods. We then 1:1 matched each ONTARGET trial participant to one trial-eligible ACEi exposed period, without replacement, on closest propensity-score, using a propensity-score model for the probability of being included in the trial (PS model 1 in Figure S2). The nearest neighbour method was used so either first or random trial-eligible ACEi exposed periods were selected depending on which was closest in propensity-score. Variables considered in the propensity score model were those available in both the ONTARGET and CPRD data and thought to be associated with trial inclusion and

outcome and based on clinical input. Variables that were likely to differ across the data sources i.e., previous medication use due to changes over time were excluded. The chosen variables were history of cerebrovascular disease, peripheral artery disease, coronary artery disease, diabetes, high-risk diabetes, age, sex, ethnicity, BMI, systolic and diastolic blood pressure and smoking status at baseline. We used a caliper of 0.25 of the standard deviation of the logit of the propensity-score, with the restriction that only one ACEi trial-eligible exposed period per patient could be matched. We used standardised differences (<0.1) and kernel density plots to assess the quality of matches which are presented in Figure S9. Matching resulted in 22,091 trial-matched ACEi patients.

**Table S1.** Key design aspects of the reference trial (ONTARGET), the target trial and deviations from protocol with emulation in CPRD GOLD data.			
**Protocol component**	**Reference trial (ONTARGET)**	**Target trial**	**Emulation in CPRD GOLD**
Eligibility criteria	Patients aged ≥55 years with coronary artery, peripheral artery or cerebrovascular disease or high-risk diabetes with end organ damage recruited up to 2004. No restriction on previous ACEi/ARB use except must be able to discontinue use.	Patients aged ≥55 years with coronary artery, peripheral artery or cerebrovascular disease or high-risk diabetes with end organ damage. No restriction on previous ACEi/ARB use except must be able to discontinue use.	Patients with a prescription for an ACE inhibitor or ARB between 01 January 2001 to 31 July 2019, eligible for HES linkage, aged ≥55 years with coronary artery, peripheral vascular, or cerebrovascular disease or high-risk diabetes.
Treatment strategies	Patients entered 3-week single blind run-in period to check compliance then randomised to one of three trial arms: ramipril 10 mg + telmisartan placebo, telmisartan 80 mg + ramipril placebo or ramipril 10 mg + telmisartan 80 mg.	Patients randomised to ACEi or ARB in a 3-week blind run-in period to check compliance then randomised to one of two trial arms: ACEi + ARB placebo or ARB + ACEi placebo	Exposure groups will be defined by prescriptions for ARBs and ACEi, respectively. Exposed periods will be consist of continuous courses of therapy with prescription gaps of <90 days making an individual exposed period.
Assignment procedures	Randomly assigned and received placebo for other drug so unaware which arm assigned to	Randomly assigned and received placebo for other drug so unaware which arm assigned to	Based on prescriptions received. Patient can contribute to both exposure groups at different timepoints
Follow-up period	Follow-up started at randomisation and ended at primary event, death, loss to follow-up or end of study. Close out was planned in July 2007.	Follow-up started at randomisation and ended at primary event, death, loss to follow-up or end of study (July 2019).	Follow-up starts at start of trial-eligible period where exposure period meets trial inclusion/exclusion criteria. Ends at the earliest of: outcome of interest, death, transferred out of practice date, or last

**Table S1.** Key design aspects of the reference trial (ONTARGET), the target trial and deviations from protocol with emulation in CPRD GOLD data.			
**Protocol component**	**Reference trial (ONTARGET)**	**Target trial**	**Emulation in CPRD GOLD**
			data collection from the general practice. If these dates do not occur the patient will be censored after 5.5 years of follow-up
Outcome	Primary composite of: cardiovascular death, MI, stroke, hospitalisation for heart failure	Primary composite of: cardiovascular death, MI, stroke, hospitalisation for heart failure	As in ONTARGET, defined using ICD10, Read codes and death registries from ONS.
Analysis plan	Primary analysis under time-to-event counting first occurrence of any component of the composite outcome using Cox proportional hazards model. Intention-to-treat as main analysis	Primary analysis under time-to-event counting first occurrence of any component of the composite outcome using Cox proportional hazards model. Intention-to-treat as main analysis	Analysis conducted on one randomly selected trial eligible period per patient. With exposed periods defined as trial eligible if trial criteria met at start of period. Balance of covariates obtained by propensity score weighting for probability of receiving an ACEi and adjusted for any imbalanced variables for main analysis. Weighting as opposed to matching to increase sample size and diversity of cohort to enable inferences to be extended to underrepresented groups. Cox proportional hazards model used for primary analysis. Propensity-score—matched analysis carried out as sensitivity.

**Table S2.** Deviations from protocol	
**Deviation**	**Reason**
Using propensity-score—weighting as opposed to propensity-score—matching (with propensity-score—matching carried out as an sensitivity analysis)	To obtain average treatment effect as opposed to average treatment effect on treated and increase sample size
Underrepresented group analysis on propensity-score—weighted sample as opposed to propensity-score—matched cohort	This was to increase sample size as comparison between both analyses gave almost identical results
Primary outcome: including both fatal and non-fatal events for stroke and myocardial infarction	Consistency with trial
Included additional outcome- main secondary outcome: composite of cardiovascular-related death, myocardial infarction, or stroke	Consistency with trial
Angina inclusion criteria: Removed condition that needed to have previous coronary artery disease diagnosis	Misclassification
CABG Inclusion criteria: Removed condition that could be with angina	Only included events where CABG was within 4 years prior to avoid due to potential of capturing old events
Comparing reason for discontinuation to safety outcomes in trial as opposed to events occurring within 3 months	Consistency with safety outcomes reported in trial
Objective of extending follow-up for safety events	Have not yet addressed this objective due to difficulty replicating safety trial results
Renal function omitted from propensity-score model	Due to large amounts of missingness
Adherence assessed differently and instead reported proportions of patients receiving each drug at different timepoints	Consistency with trial
Additional subgroups studied	To further demonstrate trial replicability and quantify effect modification
On-treatment (per-protocol) analysis for secondary objectives 1 and 2 (extending findings to trial-underrepresented and excluded groups)	Not deemed necessary as on-treatment analysis was sufficiently comparable to ITT for primary outcome
Previously mentioned that patients had to meet inclusion and exclusion criteria prior to start of first exposed period instead trial criteria assessed at start of all exposed periods	Incorrect wording in protocol this reduces bias by assessing at start of follow up
Referred to analysis group as trial-analogous now analysis groups will be labelled as propensity-score—weighted trial-eligible for main analysis and propensity-score—matched trial-eligible for sensitivity analysis	To avoid confusion as only the ACEi trial-eligible cohort is trial-matched

Naming of nephropathy outcomes	Changed from nephropathy 1 and nephropathy 2 to loss of eGFR or ESKD and ESKD
Nephropathy 1 sensitivity analysis requiring 2 measurements at least 3 months apart for both eGFR<15 and 50% reduction in eGFR	Previously stated this is only required for 50% reduction in eGFR which was incorrect

Table S3. List of variables considered and included in propensity-score model used to achieve balance across exposure groups

**Variables included in propensity-score model**	**Propensity score model for probability of receiving an ACEi**
Stroke/TIA	✓
Peripheral artery disease	✓
Coronary artery disease	✓
Diabetes	✓
High-risk diabetes	✓
Age (years)	✓
Sex	✓
Ethnicity	✓
BMI	✓
SBP	✓
DBP	✓
Index of Multiple Deprivation (IMD)	✓
Smoke status	✓
Alcohol use	✓
Statin use	✓
Nitrate use	✓
Diabetic treatment use	✓
Diuretic use	✓
CCB use	✓
Betablocker use	✓
Aspirin use	✓
Antiplatelet use	✓
Digoxin use	
Anticoagulant use	
Alpha-blocker use	
No. of hospital admissions within 6 months prior	✓
No. of GP appointments within 6 months prior	✓
No. of medications within 6 months prior	✓
Year of start of eligible period	✓
Time since first eligible period (days)	✓
No. of previous ACE inhibitor eligible periods	✓
No. of previous ARB eligible periods	✓

Notes: TIA: transient ischaemic attack; BMI: body-mass index; SBP: systolic blood pressure; DBP: diastolic blood pressure.

Variables are measured at start of trial-eligible period or before.

Peripheral artery disease includes limb bypass surgery or angioplasty, limb/foot amputation, or intermittent claudication.
Coronary artery disease includes previous MI, angina, coronary angioplasty, or CABG.
SBP and DBP are measured within 6 months prior to start of trial-eligible period.
Medication use is within 3 months prior to start of trial-eligible period.

Table S4. Explanation for potential differences in estimates from ONTARGET and the emulation in CPRD after mapping protocol components

Protocol component	Potential remaining differences	Examples	Approach to address	Result
Eligibility criteria	Differences in study population	Groups unequally represented in the emulation and ONTARGET, i.e., ONTARGET including fewer females.	Sensitivity analysis PS-matching trial-eligible ACEi patients to ONTARGET trial participants then PS-matching trial-matched ACEi patients to trial-eligible ARB periods to ensure CPRD analysis cohort has similar covariate distribution to ONTARGET	Consistent with PS-weighted approach. Main analysis (PS-weighted): HR 0.98 (95% CI: 0.94, 1.02); sensitivity analysis (PS-matched): HR 0.97 (95% CI: 0.92, 1.02)
Treatment strategies	Differences in treatment uptake	Individuals in ONTARGET may be more adherent than patients in CPRD	Compare adherence in ONTARGET to that in CPRD	Adherence similar for ARB users, differed for ACEi users with more patients in ACEi exposure group switching to an ARB
Assignment procedures	Confounding by indication	Patients who started ARB in CPRD may be healthier than those receiving an ACEi	Compared Kaplan-Meier curves in ONTARGET and emulation at 1, 2, 3, 4 and 5 years	Risk appears slightly lower for ARB users in CPRD at 1, 2, 3, 4 and 5 years compared to ONTARGET where risk is similar among telmisartan and ramipril users until 1.5 years where risk is lower among ramipril users at 2, 3 and 4 years.
Follow-up period	Differential loss to follow-up	Patients lost to follow-up in CPRD may have worse prognosis	Reanalyse as sensitivity excluding patients who were lost	Consistent with main analysis, HR 0.96 (95% CI: 0.93, 1.00)

			to follow-up in the first 12 months	
Outcome	Differences in incidence of outcome	ONTARGET was a global trial and emulation on UK population so incidence of outcome may differ by country	Include individual components of composite outcome and compare incidence between CPRD and ONTARGET	Incidence similar for most outcomes but in CPRD incidence of MI was higher than in ONTARGET

**Table S5.** Assessment of balance of variables included in propensity-score model for ARB vs ACEi analysis before and after weighting						
**Characteristic**	**Before propensity-score—weighting**			**After propensity-score—weighting**		
	**ACEi** *n=96 602*	ARB *n=40 553*	**SMD**	**ACEi** *n=145 258*	**ARB** *n=135 592*	**SMD**
**Age - year**	70.8 ± 9.0	71.2 ± 8.7	0.047	71.2 ± 8.9	71.1 ± 9.0	0.009
**Systolic BP– mmHg**	147.4 ± 20.7	148.1 ± 20.7	0.035	147.4 ± 20.3	148.2 ± 20.8	0.041
**Diastolic BP– mmHg**	80.1 ± 10.7	79.7 ± 10.5	0.041	79.5 ± 10.7	80.1 ± 10.7	0.054
**Body-mass index (kg/m^2)**	28.3 ± 5.3	28.8 ± 5.4	0.097	28.3 ± 5.7	28.6 ± 5.4	0.044
**Female sex – no. (%)**	45508 (47.1)	22690 (56.0)	0.178	753045 (51.8)	68531 (50.5)	0.026
**Ethnic group – no. (%)**						
Black	1280 (1.3)	736 (1.8)	0.039	2064 (1.4)	2053 (1.5)	0.008
Other	1134 (1.2)	607 (1.5)	0.028	1743 (1.2)	1747 (1.3)	0.008
South Asian	3026 (3.1)	1799 (4.4)	0.068	8632 (5.9)	5074 (3.7)	0.103
White	91162 (94.4)	37411 (92.3)	0.028	132819 (91.4)	126718 (93.5)	0.076
**Clinical history – no. (%)**						
CAD[a,b]	68009 (70.4)	28202 (69.5)	0.019	100441 (69.2)	94475 (69.7)	0.011
Cerebrovascular disease[a,c]	8695 (9.0)	3140 (7.7)	0.046	12627 (8.7)	11840 (8.7)	0.001
PAD[a,d]	9999 (10.4)	4078 (10.1)	0.010	14360 (9.9)	14463 (10.7)	0.026
Diabetes[e]	43751 (45.3)	20003 (49.3)	0.081	69713 (48.0)	64740 (47.8)	0.005
High-risk diabetes[a,f]	30736 (31.8)	14757 (36.4)	0.097	50470 (34.7)	46152 (34.0)	0.015
**Smoking status – no. (%)**						
Non-smoker	34503 (35.7)	16470 (40.6)	0.101	55850 (38.5)	50717 (37.4)	0.022
Current smoker	12921 (13.4)	3552 (8.8)	0.148	16660 (11.5)	16628 (12.3)	0.024
Past smoker	49178 (50.9)	20531 (50.6)	0.006	72748 (50.1)	68248 (50.3)	0.005
**Alcohol status – no. (%)**						
Drinker	76756 (79.5)	31840 (78.5)	0.023	116186 (80.0)	106324 (78.4)	0.039
**Medication[g] – no. (%)**						

Table S5. Assessment of balance of variables included in propensity-score model for ARB vs ACEi analysis before and after weighting						
**Characteristic**	**Before propensity-score—weighting**			**After propensity-score—weighting**		
	**ACEi** *n=96 602*	ARB *n=40 553*	**SMD**	**ACEi** *n=145 258*	**ARB** *n=135 592*	**SMD**
Antiplatelet agent	12334 (12.8)	2482 (6.1)	0.229	14650 (10.1)	13132 (9.7)	0.014
Aspirin	44011 (45.6)	9325 (23.0)	0.489	53049 (36.5)	50799 (37.5)	0.020
Beta-blocker	34178 (35.4)	6756 (16.7)	0.437	40752 (28.1)	38741 (28.6)	0.012
Calcium-channel blocker	28820 (29.8)	8515 (21.0)	0.204	38254 (26.3)	37865 (27.9)	0.036
Diuretics	32002 (33.1)	8838 (21.8)	0.256	40789 (28.1)	41888 (30.9)	0.062
Diabetic treatment	20060 (20.8)	4910 (12.1)	0.235	25258 (17.4)	25562 (18.9)	0.038
Nitrates	14862 (15.4)	3172 (7.8)	0.238	17722 (12.2)	16577 (12.2)	0.001
Statins	52925 (54.8)	11474 (28.3)	0.558	64630 (44.5)	61780 (45.6)	0.021
**Index of multiple deprivation (IMD) – no.(%)**						
1 (least deprived)	19805 (20.5)	8996 (22.2)	0.041	32569 (22.4)	28190 (20.8)	0.040
2	21977 (22.8)	9603 (23.7)	0.022	34344 (23.6)	30971 (22.8)	0.019
3	20644 (21.4)	8674 (21.4)	0.001	30256 (20.8)	28912 (21.3)	0.012
4	18462 (19.1)	7312 (18.0)	0.028	26032 (17.9)	25280 (18.6)	0.019
5 (most deprived)	15714 (16.3)	5967 (14.7)	0.043	22057 (15.2)	22239 (16.4)	0.033
**Health utilisation**[h]						
No. of hospital admissions	0.42 ± 1.0	0.31 ± 0.9	0.118	0.38 ± 0.9	0.36 ± 0.9	0.017
No. of GP apt.	27.9 ± 27.3	15.2 ± 25.4	0.482	22.8 ± 26.9	24.3 ± 29.5	0.058
No. of different drug types	8.5 ± 4.3	9.7 ± 4.6	0.273	9.2 ± 4.5	9.0 ± 4.6	0.035
**Time-related variables**						
Time since first eligible period (days)	131.3 ± 496.5	377.6 ± 759.1	0.384	544.7 ± 1444.4	223.6 ± 625.4	0.501
No. of prior ARB periods	0.04 ± 0.3	0.1 ± 0.5	0.163	0.1 ± 0.4	0.1 ± 0.4	0.117
No. of prior ACEi periods	0.1 ± 0.5	0.6 ± 0.7	0.739	0.3 ± 0.8	0.3 ± 0.6	0.053
Calendar year	2007.1 ± 4.0	2007.6 ± 4.1	0.131	2007.9 ± 4.6	2007.3 ± 4.1	0.152

**Table S5.** Assessment of balance of variables included in propensity-score model for ARB vs ACEi analysis before and after weighting						
**Characteristic**	**Before propensity-score—weighting**			**After propensity-score—weighting**		
	**ACEi** *n=96 602*	ARB *n=40 553*	**SMD**	**ACEi** *n=145 258*	**ARB** *n=135 592*	**SMD**

Notes: SMD=standardised mean difference; BP=blood pressure.

Post-weighting n displays weighted distribution of number of patients in the two exposure groups.

Propensity-score weights are unstabilized inverse probability weights.

Cohort includes 1 randomly selected eligible period per patient in each group

[a] Any diagnosis prior to start of eligible period

[b] Includes diagnosis of: MI at least 2 days prior, angina at least 30 days prior, angioplasty at least 30 days prior, CABG at least 4 years prior

[c] Includes diagnosis of: stroke/TIA

[d] Includes diagnosis of: limb bypass surgery, limb/foot amputation, intermittent claudication

[e] DM prior to start of eligible period

[f] Includes DM with: retinopathy, neuropathy, chronic kidney disease or proteinuria

[g] Within 3 months prior to eligible start date. Antiplatelet agent= clopidogrel/ ticlopidine

[h] Within 6 months prior to eligible start date.

no. (%)=number (percent); CAD=coronary artery disease; MI=myocardial infarction; PAD=peripheral artery disease.

**Table S6.** Medication adherence to assigned exposure group				
**Years of follow-up**	**ACEi patients** *n=96 602*		**ARB patients** *n=40 553*	
	**Receiving**		**Receiving**	
	**ACEi**	**ARB**	**ARB**	**ACEi**
1	67347 (69.7)	10805 (11.2)	31661 (78.1)	1039 (2.6)
2	57188 (59.2)	12177 (12.6)	27381 (67.5)	1483 (3.7)
3	48991 (50.7)	12148 (12.6)	23487 (57.9)	1718 (4.2)
4	41583 (43.1)	11523 (11.9)	19950 (49.2)	1828 (4.5)
5.5	31769 (32.9)	10060 (10.4)	15210 (37.5)	1764 (4.4)

Table S7. Secondary and other outcomes after propensity-score—weighted analysis using CPRD data

Outcome	ACEi<br>*n=96 602*	ARB<br>*n=40 553*	ARB vs ACEi
	Number (percent)		*Hazard ratio (95% CI)*
Newly diagnosed congestive heart failure	10232 (10.6)	4017 (9.9)	0.99 (0.94, 1.04)
Revascularisation procedures	14132 (14.6)	5250 (13.0)	1.00 (0.96, 1.04)
Loss of GFR or ESKD	4217 (5.2)	2205 (6.1)	1.11 (1.04, 1.19)
ESKD	1460 (1.8)	822 (2.3)	1.06 (0.95, 1.19)
Microvascular complications of diabetes mellitus	2261 (17.4)	757 (14.4)	0.95 (0.85, 1.05)

Notes: ESKD: end-stage kidney disease.

CPRD weighted analysis includes 1 randomly selected eligible period per patient. Propensity-score—weighted with robust standard errors. ARB vs ACEi also adjusted for time since first eligible period, calendar year and number of prior ARB periods.

Loss of GFR or ESKD defined as: 50% reduction in estimated glomerular filtration ratio (eGFR), start of kidney replacement therapy (KRT) or eGFR<15.

ESKD defined as: start of KRT or eGFR<15.

Kidney outcomes only include those subjects who have an eGFR measurement before start of the eligible period but within 6 months and adjusted for baseline serum creatinine.

Microvascular complications of diabetes mellitus outcome only include patients who are diabetic but non-high risk.

Table S8. Reason for treatment cessation using trial criteria and propensity-score—weighting for ARB vs ACEi compared to ONTARGET

Reason for cessation	CPRD			ONTARGET
	**ARB** *n=40 553*	**ACEi** *n=96 602*	**ARB vs ACEi**	**Telmisartan vs ramipril**
	Number (percent)		*Relative risk (95% CI)*	*Relative risk (P value)*
Cough	949 (2.3)	1557 (1.6)	1.29 (1.16, 1.43)	0.26 (<0.001)
Angioedema	37 (0.09)	83 (0.09)	1.14 (0.72, 1.80)	0.4 (0.01)
Hyperkalaemia[a]	2784 (7.8)	5836 (7.4)	1.12 (1.06, 1.18)	
≥30% increase in serum creatinine	7222 (19.8)	12441 (15.2)	1.38 (1.34, 1.43)	1.14 (0.46)[b]

Notes: In CPRD, treatment cessation is defined as the end date of the trial-eligible exposed period included in analysis (i.e., the date prior to a prescription gap of >90 days) and the latest event occurring prior to end of trial-eligible period is counted as the reason for treatment cessation. Multiple reasons that occur on the same day are both counted.

Analysis is adjusted for time since first eligible period, calendar year and number of prior ARB eligible periods.

[a]Defined as potassium >5.5 mmol/l. Analysis out of number of people with non-missing potassium.

[b]Definition of renal impairment as reason for discontinuation in ONTARGET is not stated so results are not directly comparable to CPRD

Kidney outcomes are adjusted for baseline serum creatinine and are out of the number of people with non-missing eGFR in CPRD.

ONTARGET did not present 95% CI.

Table S9. Assessment of balance of variables included in propensity-score model for ARB vs ACEi sensitivity analysis after matching compared to ONTARGET				
**Characteristic**	**Propensity-score—matched analysis cohort**			**ONTARGET**
	**ACEi** *n=15,462*	**ARB** *n=15,462*	**SMD**	*n=25620*
**Age - year**	67.4 ± 8.3	67.8 ± 8.4	0.047	66.4 ± 7.2
**Systolic BP– mmHg**	143.1 ± 17.7	143.8 ± 17.9	0.035	141.8 ± 17.4
**Diastolic BP– mmHg**	82.1 ± 10.3	81.8 ± 10.3	0.028	82.1 ± 10.4
**Body-mass index (kg/m^2)**	28.6 ± 5.2	28.8 ± 5.3	0.037	28.2 ± 4.7
**Female sex – no. (%)**	5026 (32.5)	5554 (35.9)	0.072	6831 (26.7)
**Ethnic group – no. (%)**				
Black	409 (2.6)	429 (2.8)	0.008	629 (2.5)
Other	623 (4.0)	483 (3.1)	0.049	4901 (19.1)
South Asian	922 (6.0)	913 (5.9)	0.002	1375 (5.4)
Unknown	-	-	-	7 (<0.1)
White	13508 (87.4)	13637 (88.2)	0.025	18708 (73.0)
**Clinical history – no. (%)**				
CAD[a,b]	11474 (74.2)	11275 (72.9)	0.029	19102 (74.6)
Cerebrovascular disease[a,c]	2287 (14.8)	1902 (12.3)	0.073	5342 (20.9)
PAD[a,d]	1992 (12.9)	1856 (12.0)	0.027	3468 (13.5)
Diabetes[e]	6255 (40.5)	6647 (43.0)	0.051	9612 (37.5)
High-risk diabetes[a,f]	4675 (30.2)	4947 (32.0)	0.038	7151 (27.9)
**Smoking status – no. (%)**				
Non-smoker	5666 (36.6)	5799 (37.5)	0.019	9088 (35.5)
Current smoker	1765 (11.4)	1655 (10.7)	0.022	3225 (12.6)
Past smoker	8031 (51.9)	8008 (51.8)	0.002	13276 (51.8)
**Alcohol status – no. (%)**				
Drinker	12421 (80.3)	12317 (79.7)	0.017	10345 (40.4)
**Medication[g] – no. (%)**				
Antiplatelet agent	1875 (12.1)	1577 (10.2)	0.061	2824 (11.0)
Aspirin	5987 (38.7)	5685 (36.8)	0.040	19403 (75.7)
Beta-blocker	4429 (28.6)	4169 (27.0)	0.038	14583 (56.9)
Calcium-channel blocker	4010 (25.9)	4115 (26.6)	0.015	8472 (33.1)
Diuretics	4128 (26.7)	4223 (27.3)	0.014	7164 (28.0)
Diabetic treatment	2615 (16.9)	2735 (17.7)	0.021	8056 (31.4)

Table S9. Assessment of balance of variables included in propensity-score model for ARB vs ACEi sensitivity analysis after matching compared to ONTARGET

Characteristic	Propensity-score—matched analysis cohort			ONTARGET
	ACEi *n=15,462*	ARB *n=15,462*	SMD	*n=25620*
Nitrates	1936 (12.5)	1761 (11.4)	0.035	7523 (29.4)
Statins	7450 (48.2)	7128 (46.1)	0.042	15783 (61.6)
**Index of multiple deprivation (IMD) – no.(%)**				
1 (least deprived)	3237 (20.9)	3358 (21.7)	0.020	
2	3474 (22.5)	3525 (22.8)	0.007	
3	3330 (21.5)	3293 (21.3)	0.005	
4	2932 (19.0)	2902 (18.8)	0.005	
5 (most deprived)	2489 (16.1)	2384 (15.4)	0.019	
**Health utilisation**[h]				
No. of hospital admissions	0.40 ± 0.87	0.36 ± 0.90	0.040	
No. of GP apt.	23.2 ± 26.4	23.4 ± 27.9	0.006	
No. of different drug types	8.5 ± 4.5	8.7 ± 4.5	0.045	
**Time-related variables**				
Time since first eligible period (days)	241.1 ± 628.0	241.0 ± 631.7	0.001	
No. of prior ARB periods	0.07 ± 0.32	0.04 ± 0.27	0.089	
No. of prior ACEi periods	0.30 ± 0.79	0.29 ± 0.66	0.010	

Notes: SMD=standardised mean difference; BP=blood pressure.

Cohort includes 1 randomly selected eligible period per patient in each group

[a] Any diagnosis prior to start of eligible period

[b] Includes diagnosis of: MI at least 2 days prior, angina at least 30 days prior, angioplasty at least 30 days prior, CABG at least 4 years prior

[c] Includes diagnosis of: stroke/TIA

[d] Includes diagnosis of: limb bypass surgery, limb/foot amputation, intermittent claudication

[e] DM prior to start of eligible period

[f] Includes DM with: retinopathy, neuropathy, chronic kidney disease or proteinuria

[g] Within 3 months prior to eligible start date. Antiplatelet agent= clopidogrel/ ticlopidine

[h] Within 6 months prior to eligible start date.

**Table S9.** Assessment of balance of variables included in propensity-score model for ARB vs ACEi sensitivity analysis after matching compared to ONTARGET				
**Characteristic**	**Propensity-score—matched analysis cohort**			**ONTARGET** *n=25620*
	**ACEi** *n=15,462*	**ARB** *n=15,462*	**SMD**	
no. (%)=number (percent); CAD=coronary artery disease; MI=myocardial infarction; PAD=peripheral artery disease.				

Table S10. Number of events in the primary outcome, its components, and death from any cause for ARB vs ACEi using a propensity-score—matched analysis of patients in CPRD (sensitivity analysis)

**Outcome**	**CPRD: Propensity-score—matched**			**ONTARGET**
	**ACEi** *n=15 462*	**ARB** *n=15 462*	**ARB vs ACEi**	**Telmisartan vs Ramipril**
	Number (percent)		*Hazard ratio (95% CI)*	
Primary composite: Death from cardiovascular causes, myocardial infarction, stroke, or hospitalisation for heart failure	2539 (16.4)	2453 (15.9)	0.97 (0.92, 1.02)	1.01 (0.94, 1.09)
Main secondary outcome: Death from cardiovascular causes, myocardial infarction, or stroke	2234 (14.5)	2173 (14.1)	0.98 (0.92, 1.04)	0.99 (0.91, 1.07)
Myocardial infarction	1806 (11.7)	1721 (11.1)	0.96 (0.90, 1.03)	1.07 (0.94, 1.22)
Stroke	535 (3.5)	591 (3.8)	1.10 (0.98, 1.24)	0.91 (0.79, 1.05)
Hospitalisation for heart failure	542 (3.5)	513 (3.3)	0.94 (0.83, 1.06)	1.12 (0.97, 1.29)
Death from cardiovascular causes	655 (4.2)	649 (4.2)	0.98 (0.88, 1.09)	1.00 (0.89, 1.12)
Death from non-cardiovascular causes	852 (5.5)	856 (5.5)	0.99 (0.90, 1.09)	0.96 (0.83, 1.10)
Death from any cause	1507 (9.8)	1505 (9.7)	0.99 (0.92, 1.06)	0.98 (0.90, 1.07)

Notes: Propensity-score—matched cohort developed using trial-matched ACEi patients 1:1 matched to closest trial-eligible ARB period.

Myocardial infarction and stroke include both fatal and non-fatal events.

ONTARGET results are from published findings.

Table S11. Safety outcomes assessed among non-switchers using trial criteria and propensity-score—weighting for ARB vs ACEi (sensitivity analysis)

**Safety outcome**	**CPRD**			**ONTARGET**
	**Reason for treatment cessation**			
	**ARB** *n=11 856*	**ACEi** *n=90 597*	**ARB vs ACEi**	**Telmisartan vs ramipril**
	Number (percent)		*Relative risk (95% CI)*	*Relative risk (P value)*
Cough	178 (1.5)	1455 (1.6)	0.84 (0.70, 1.01)	0.26 (<0.001)
Angioedema	10 (0.08)	77 (0.08)	0.72 (0.35, 1.48)	0.4 (0.01)
Hyperkalaemia[a]	685 (7.7)	5473 (7.4)	1.06 (0.97, 1.16)	-
≥30% increase in serum creatinine	1730 (18.8)	11781 (15.5)	1.25 (1.18, 1.31)	1.14 (0.01)[b]

Notes: In CPRD, treatment cessation is defined as the end date of the trial-eligible exposed period included in analysis (i.e., the date prior to a prescription gap of >90 days) and the latest event occurring prior to end of trial-eligible period is counted as the reason for treatment cessation. Multiple reasons that occur on the same day are both counted.

Reason for treatment cessation represents the main analysis which is compared to ONTARGET and events occurring within 3 months represents the additional analysis exploring safety events which occur within 3 months of the start of eligible period.

Non-switchers include first trial-eligible period per patient and excludes patients with previous exposure to opposing drug at any time prior to start of included trial-eligible period.

Both analyses are adjusted for number of previous GP appointments within 6 months prior.

[a]Defined as potassium >5.5 mmol/l. Analysis out of number of people with non-missing potassium.

[b]Definition of renal impairment in ONTARGET is not stated so results are not directly comparable to CPRD

Kidney outcomes are adjusted for baseline serum creatinine and are out of the number of people with non-missing eGFR in CPRD.

ONTARGET did not present 95% CI.

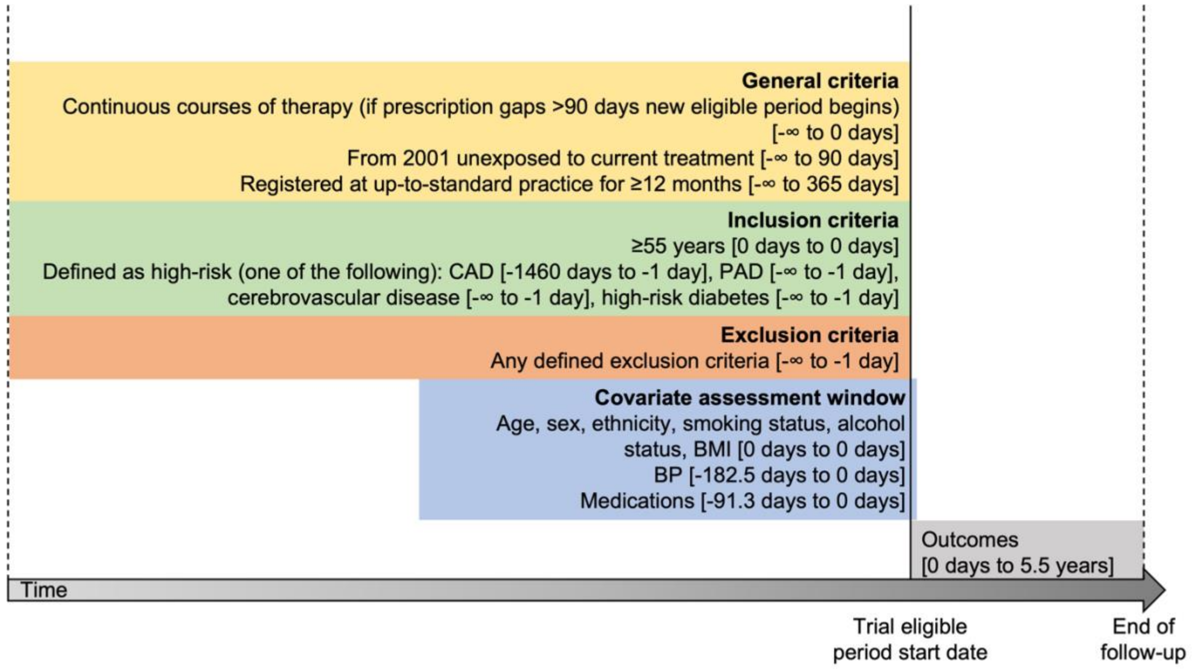

Figure S1. Study diagram

End of follow-up was earliest of date of outcome, transferred out of practice date, death date, date of last collection, or 5.5 years from the start of eligible period. Trial eligible periods are defined as exposed periods where all trial criteria are met prior to the start of the exposed period. An exposed period is defined as periods of continuous courses of therapy (<90 days between prescriptions). CAD=coronary artery disease, PAD=peripheral artery disease. Details of how inclusion and exclusion criteria were defined are published previously. An up-to-standard practice is one that meets minimum data quality criteria based on continuity of recording and recorded number of deaths.

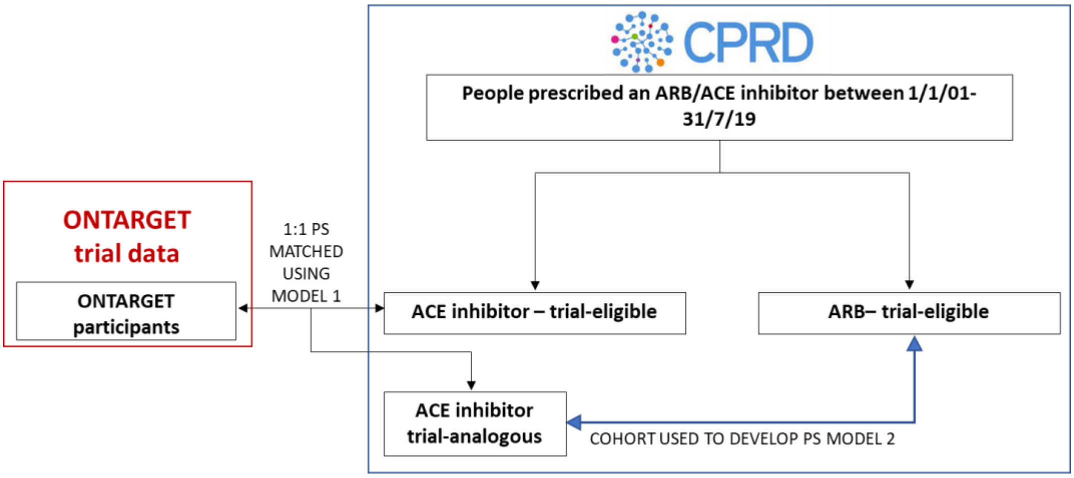

Figure S2. Steps to develop propensity score models used to achieve balance across CPRD exposure groups. PS=propensity-score; PS model 1: probability of being in the reference trial (ONTARGET); PS model 2: probability of receiving an ACEi

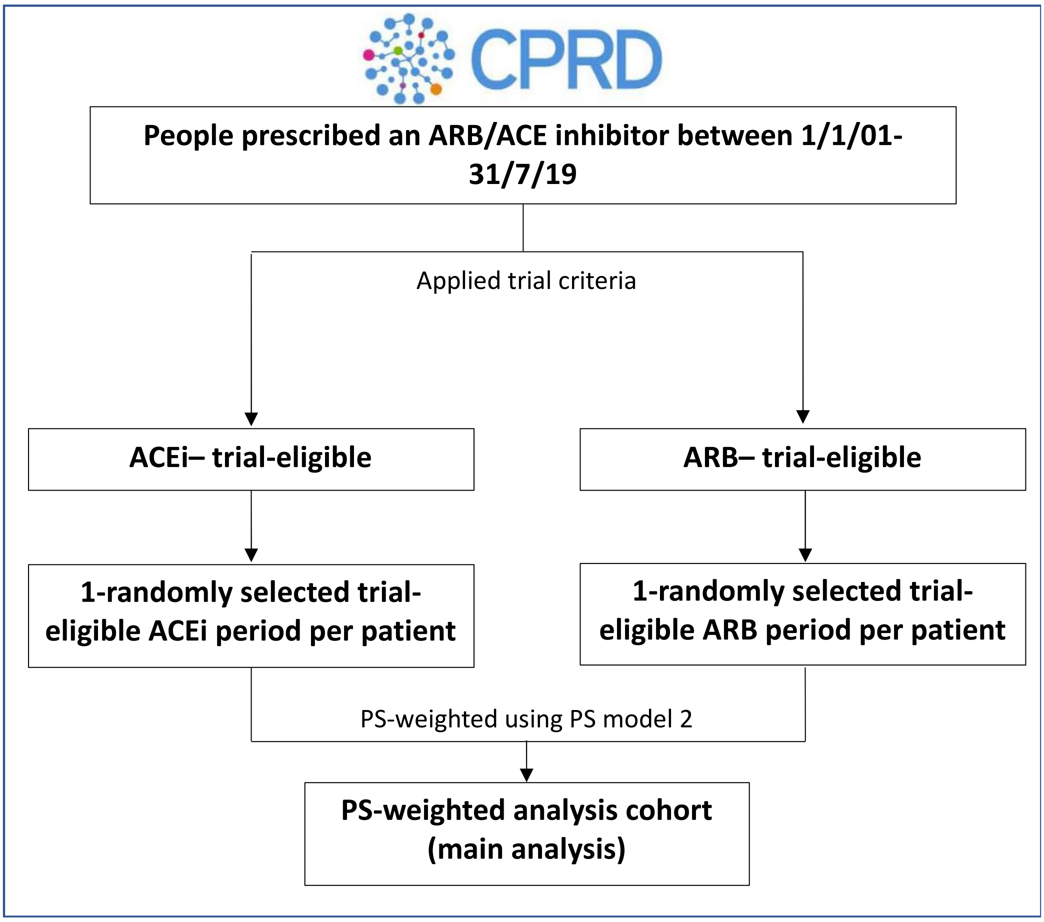

Figure S3. Steps to achieve balance across exposure groups in main analysis using propensity-score—weighting. PS=propensity-score; PS model 2: probability of receiving an ACEi

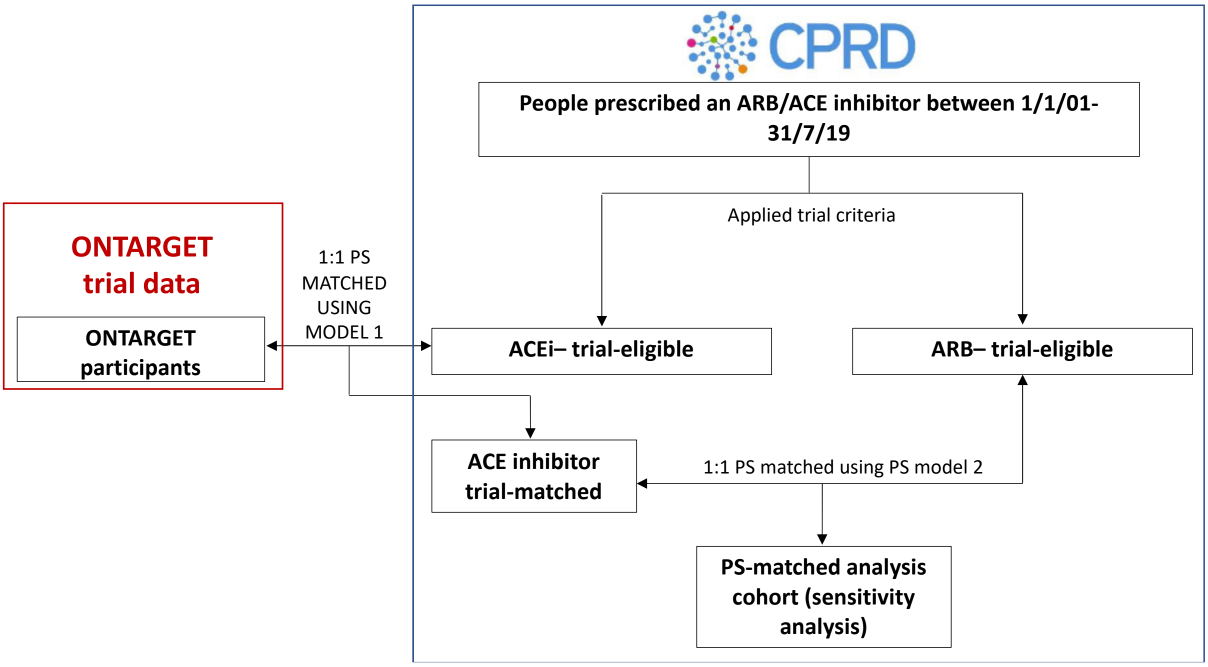

Figure S4. Steps to achieve balance across exposure groups in sensitivity analysis using propensity-score—matching. PS=propensity-score; PS model 1: probability of being in the reference trial; PS model 2: probability of receiving an ACEi

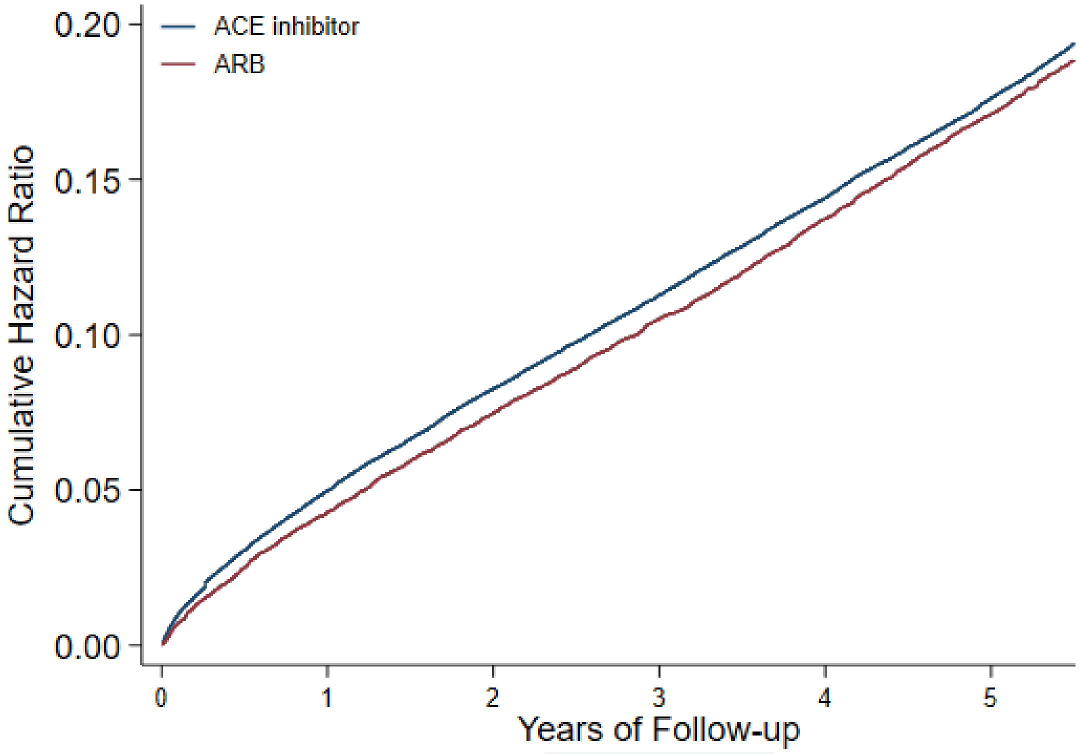

Figure S5. Kaplan-Meier curves for the Primary composite outcome for ARB and ACEi users.

Outcome	ARB (N=40,553) n (%)	ACEi (N=96,602) n (%)	ARB vs ACEi HR (95% CI)	P Value for Interaction
Primary composite				0.21
Male	3166 (17.7)	9931 (19.4)	0.94 (0.88, 1.00)	
Female	3121 (13.8)	7004 (15.4)	1.00 (0.94, 1.06)	
Main secondary				0.37
Male	2746 (15.4)	8747 (17.1)	0.95 (0.89, 1.01)	
Female	2617 (11.5)	5900 (13.0)	1.00 (0.93, 1.07)	
Myocardial infarction				0.25
Male	2228 (12.4)	7344 (14.4)	0.92 (0.86, 0.99)	
Female	1862 (8.2)	4273 (9.4)	0.99 (0.91, 1.08)	
Stroke				0.14
Male	665 (3.7)	1811 (3.5)	1.10 (0.98, 1.23)	
Female	908 (4.0)	1957 (4.3)	0.98 (0.89, 1.08)	
Hospitalisation for HF				0.81
Male	761 (4.3)	2158 (4.2)	0.96 (0.86, 1.07)	
Female	809 (3.6)	1870 (4.1)	0.94 (0.84, 1.05)	
Death from CV causes				0.03
Male	897 (5.0)	2791 (5.5)	1.02 (0.93, 1.12)	
Female	928 (4.1)	2403 (5.3)	0.88 (0.80, 0.97)	
Death from non-CV causes				0.32
Male	1223 (6.8)	3665 (7.2)	0.98 (0.91, 1.07)	
Female	1426 (6.3)	3319 (7.3)	0.93 (0.86, 1.00)	
Death from any cause				0.04
Male	2120 (11.9)	6456 (12.6)	1.00 (0.94, 1.06)	
Female	2354 (10.4)	5722 (12.6)	0.91 (0.85, 0.97)	
Newly diagnosed HF				0.62
Male	1944 (10.9)	5649 (11.1)	0.96 (0.90, 1.02)	
Female	2073 (9.1)	4583 (10.1)	0.98 (0.92, 1.05)	
Revascularisation proc				0.03
Male	3397 (19.0)	10185 (19.9)	0.96 (0.91, 1.01)	
Female	1853 (8.2)	3947 (8.7)	1.06 (0.98, 1.15)	
Loss of GFR or ESKD				0.32
Male	965 (6.0)	2026 (4.8)	1.13 (1.03, 1.25)	
Female	1240 (6.1)	2191 (5.5)	1.06 (0.97, 1.16)	
ESKD				0.79
Male	380 (2.4)	718 (1.7)	1.06 (0.90, 1.25)	
Female	442 (2.2)	742 (1.9)	1.03 (0.88, 1.20)	
Microvascular comps of DM				0.80
Male	428 (17.1)	1455 (19.6)	0.92 (0.80, 1.06)	
Female	329 (12.0)	806 (14.4)	0.95 (0.80, 1.12)	

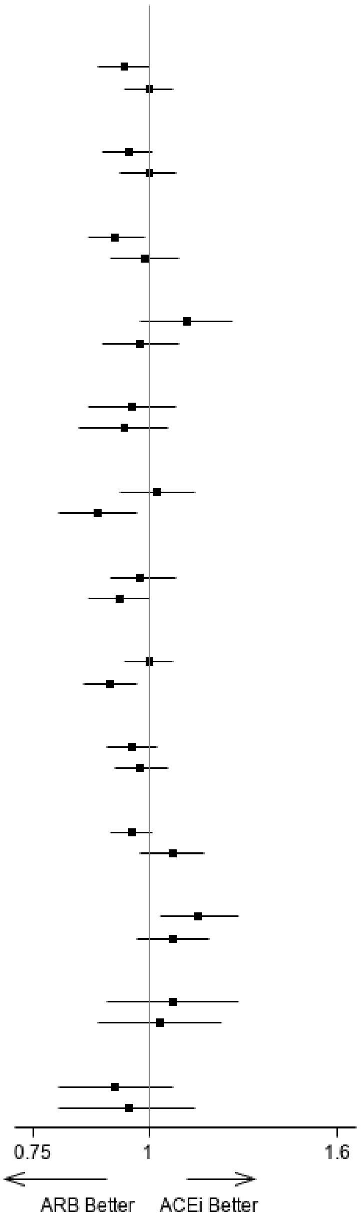

Figure S6. Treatment heterogeneity by sex for all outcomes for comparison of ARB vs ACEi.

n (%)= number of events (percent). P-value is the test of interaction between the treatment for each outcome. ESKD: end-stage kidney disease. Analysis is propensity-score—weighted with robust standard errors. Analysis adjusted for number of previous GP appointments and medications within 6 months prior, time since first eligible period and number of prior ARB periods. Loss of GFR or ESKD defined as: 50% reduction in estimated glomerular filtration rate (GFR), start of kidney replacement therapy (KRT) or eGFR<15. ESKD defined as: start of KRT or eGFR<15. Kidney outcomes only include those subjects who have an eGFR measurement before start of the eligible period but within 6 months and adjusted for baseline serum creatinine. Microvascular complications of diabetes mellitus outcome only include patients who are diabetic but non-high risk.

Outcome	ARB (N=36,919) n (%)	ACEi (N=83,515) n (%)	ARB vs ACEi HR (95% CI)	P Value for Interaction
Primary composite				0.62
<65 yr	1127 (11.1)	3625 (13.4)	0.99 (0.91, 1.08)	
>=65 to <75 yr	2096 (13.6)	5489 (15.7)	0.97 (0.90, 1.05)	
>=75 yr	3064 (20.3)	7821 (22.6)	0.94 (0.89, 1.00)	
Main secondary				0.64
<65 yr	1023 (10.1)	3350 (12.3)	1.00 (0.92, 1.10)	
>=65 to <75 yr	1809 (11.8)	4831 (13.8)	0.97 (0.89, 1.05)	
>=75 yr	2531 (16.8)	6466 (18.7)	0.95 (0.89, 1.02)	
Myocardial infarction				0.73
<65 yr	870 (8.6)	2973 (11.0)	0.99 (0.89, 1.09)	
>=65 to <75 yr	1413 (9.2)	3922 (11.2)	0.95 (0.86, 1.05)	
≥75 yr	1807 (12.0)	4722 (13.7)	0.94 (0.86, 1.02)	
Stroke				0.45
<65 yr	188 (1.9)	478 (1.8)	1.16 (0.94, 1.43)	
>=65 to <75 yr	494 (3.2)	1142 (3.3)	1.01 (0.88, 1.16)	
>=75 yr	891 (5.9)	2148 (6.2)	1.00 (0.91, 1.10)	
Hospitalisation for HF				0.79
<65 yr	187 (1.8)	489 (1.8)	0.99 (0.78, 1.25)	
>=65 to <75 yr	486 (3.2)	1201 (3.4)	0.97 (0.85, 1.11)	
>=75 yr	897 (5.9)	2338 (6.8)	0.93 (0.84, 1.02)	
Death from CV causes				0.89
<65 yr	153 (1.5)	455 (1.7)	0.99 (0.79, 1.25)	
>=65 to <75 yr	454 (3.0)	1275 (3.7)	0.93 (0.81, 1.06)	
>=75 yr	1218 (8.1)	3464 (10.0)	0.94 (0.87, 1.02)	
Death from non-CV causes				0.23
<65 yr	261 (2.6)	669 (2.5)	1.07 (0.90, 1.28)	
>=65 to <75 yr	803 (5.2)	1969 (5.6)	0.97 (0.88, 1.08)	
>=75 yr	1585 (10.5)	4346 (12.6)	0.91 (0.85, 0.99)	
Death from any cause				0.32
<65 yr	414 (4.1)	1124 (4.1)	1.04 (0.90, 1.20)	
>=65 to <75 yr	1257 (8.2)	3244 (9.3)	0.96 (0.88, 1.04)	
>=75 yr	2803 (18.6)	7810 (22.6)	0.93 (0.88, 0.98)	
Newly diagnosed HF				0.50
<65 yr	505 (5.0)	1494 (5.5)	0.91 (0.79, 1.04)	
>=65 to <75 yr	1276 (8.3)	3171 (9.1)	0.96 (0.88, 1.04)	
>=75 yr	2236 (14.8)	5567 (16.1)	0.99 (0.93, 1.05)	
Revascularisation proc				0.02
<65 yr	1373 (13.6)	4669 (17.2)	0.91 (0.84, 0.98)	
>=65 to <75 yr	2219 (14.4)	5637 (16.1)	1.04 (0.97, 1.12)	
>=75 yr	1658 (11.0)	3826 (11.1)	1.03 (0.95, 1.12)	
Loss of GFR or ESKD				0.00
<65 yr	434 (4.8)	675 (3.1)	1.42 (1.22, 1.66)	
>=65 to <75 yr	745 (5.4)	1426 (4.8)	1.09 (0.97, 1.22)	
>=75 yr	1026 (7.5)	2116 (7.0)	1.00 (0.91, 1.10)	
ESKD				0.06
<65 yr	197 (2.2)	266 (1.2)	1.28 (1.00, 1.63)	
>=65 to <75 yr	291 (2.1)	495 (1.7)	1.12 (0.92, 1.37)	
>=75 yr	334 (2.4)	699 (2.3)	0.91 (0.77, 1.07)	
Microvascular comps of DM				0.24
<65 yr	282 (16.6)	921 (19.9)	0.96 (0.81, 1.13)	
>=65 to <75 yr	352 (15.6)	954 (17.8)	0.99 (0.84, 1.15)	
>=75 yr	123 (9.5)	386 (12.7)	0.76 (0.59, 0.99)	

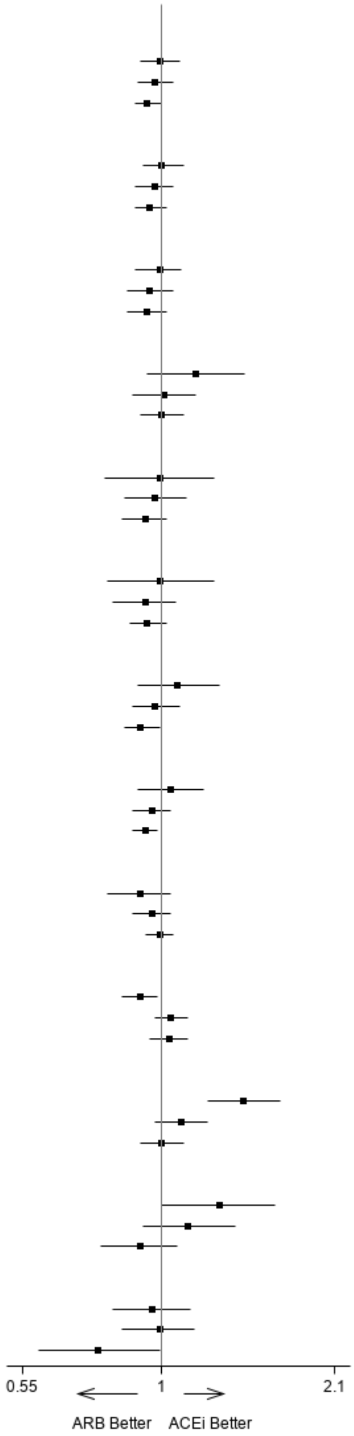

Figure S7. Treatment heterogeneity by age group for comparison of ARB vs ACEi.
P-value is the test of interaction between the treatment for each outcome. ESKD: end-stage kidney disease. Propensity-score—weighted analysis with robust standard errors and adjusted for number of medications within 6 months prior, time since first eligible period, number of prior ARB and ACEi periods, age and DBP. Loss of GFR or ESKD: 50% reduction in estimated glomerular filtration rate (eGFR), start of kidney replacement therapy (KRT) or eGFR<15. ESKD: start of KRT or eGFR<15. Kidney outcomes include subjects who have an eGFR measurement within 6 months prior to the start of the eligible period and adjusted for baseline serum creatinine. Microvascular comps of DM includes diabetics but non-high risk.

Outcome	ARB (N=36,919) n (%)	ACEi (N=83,515) n (%)	ARB vs ACEi HR (95% CI)	P Value for Interaction
Primary composite				0.17
No CKD	3126 (13.3)	8417 (15.2)	0.94 (0.89, 0.99)	
CKD	2484 (18.4)	5553 (19.6)	1.00 (0.94, 1.06)	
Main secondary				0.06
No CKD	2716 (11.6)	7405 (13.4)	0.94 (0.88, 1.00)	
CKD	2058 (15.3)	4556 (16.1)	1.03 (0.96, 1.10)	
Myocardial infarction				0.02
No CKD	2139 (9.1)	5973 (10.8)	0.92 (0.86, 0.99)	
CKD	1482 (11.0)	3310 (11.7)	1.05 (0.97, 1.14)	
Stroke				0.67
No CKD	709 (3.0)	1782 (3.2)	0.97 (0.86, 1.09)	
CKD	705 (5.2)	1516 (5.4)	1.00 (0.90, 1.12)	
Hospitalisation for HF				0.68
No CKD	676 (2.9)	1758 (3.2)	0.91 (0.80, 1.02)	
CKD	740 (5.5)	1694 (6.0)	0.94 (0.84, 1.05)	
Death from CV causes				0.21
No CKD	697 (3.0)	2109 (3.8)	0.87 (0.78, 0.97)	
CKD	966 (7.2)	2464 (8.7)	0.96 (0.87, 1.05)	
Death from non-CV causes				0.81
No CKD	1152 (4.9)	3159 (5.7)	0.95 (0.87, 1.03)	
CKD	1290 (9.6)	3065 (10.8)	0.96 (0.88, 1.04)	
Death from any cause				0.33
No CKD	1849 (7.9)	5268 (9.5)	0.92 (0.85, 0.98)	
CKD	2256 (16.7)	5529 (19.5)	0.96 (0.90, 1.02)	
Newly diagnosed HF				0.03
No CKD	1774 (7.6)	4798 (8.7)	0.91 (0.84, 0.98)	
CKD	1859 (13.8)	4037 (14.3)	1.02 (0.95, 1.10)	
Revascularisation proc				0.01
No CKD	3096 (13.2)	8370 (15.2)	0.95 (0.89, 1.00)	
CKD	1616 (12.0)	3145 (11.1)	1.08 (0.99, 1.17)	
Loss of GFR or ESKD				0.85
No CKD	945 (4.0)	2047 (3.7)	1.11 (1.00, 1.23)	
CKD	1404 (10.4)	2420 (8.5)	1.10 (0.99, 1.21)	
ESKD				0.39
No CKD	176 (0.8)	418 (0.8)	1.00 (0.80, 1.25)	
CKD	789 (5.8)	1253 (4.4)	1.13 (0.96, 1.32)	

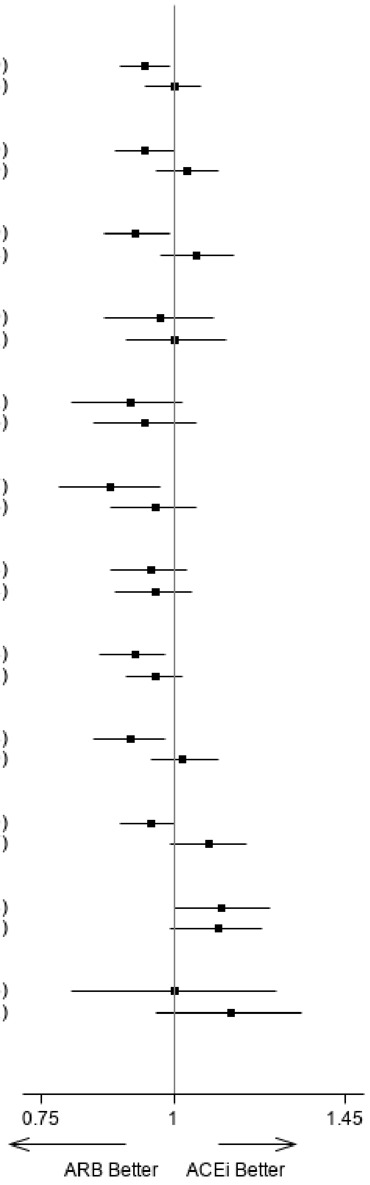

Figure S8. Treatment heterogeneity by CKD status for all outcomes for comparison of ARB vs ACEi.

CKD: estimated GFR <60 mL/min/1.73m^2; ESKD: end-stage kidney disease. n (%)= number of events (percent). P-value is the test of interaction between the treatment for each outcome. Analysis is propensity-score—weighted with robust standard errors. Analysis adjusted for time since first eligible period. Loss of GFR or ESKD defined as: 50% reduction in estimated glomerular filtration rate (eGFR), start of kidney replacement therapy (KRT) or eGFR<15. ESKD defined as: start of KRT or eGFR<15. Kidney outcomes only include those subjects who have an eGFR measurement before start of the eligible period but within 6 months and adjusted for baseline serum creatinine.

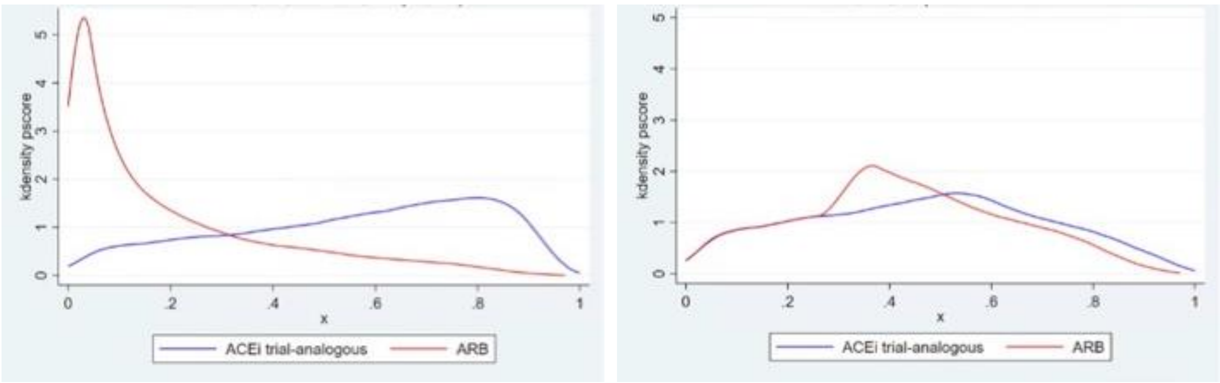

Figure S9. Kernel density plots before and after matching trial-matched ACEi patients to trial-eligible ARB patients. Left: before matching; right: after matching.

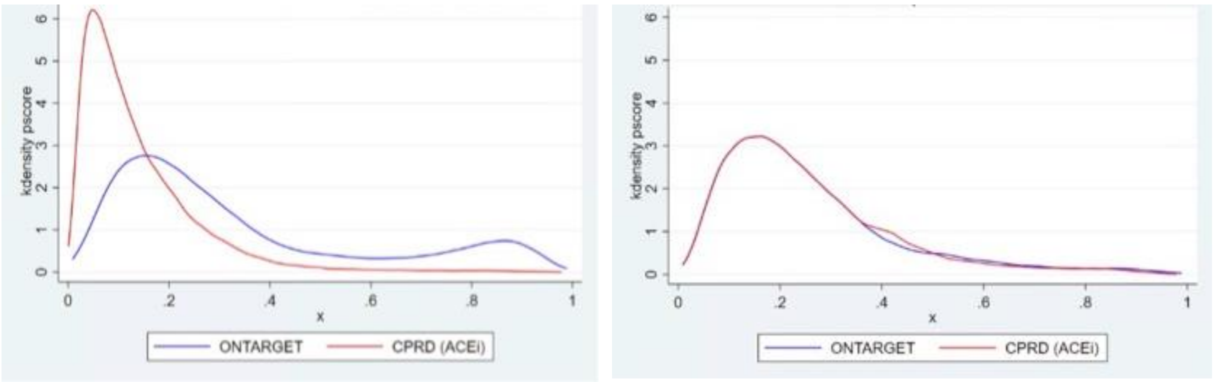

Figure S10. Kernel density plots before and after matching ONTARGET participants to trial-eligible ACEi patients. Left: before matching; right: after matching.
